# Multi-Scale Attention Conditional Domain Adaptation for Electric Control Valve Fault Diagnosis Under Variable Working Conditions

**DOI:** 10.3390/s26175524

**Published:** 2026-08-31

**Authors:** Talatibieke Aierken, Shuxun Li, Kang Yuan, Yu Zhao

**Affiliations:** 1 School of Petrochemical Engineering, Lanzhou University of Technology, Lanzhou 730050, China; bieke18@163.com (T.A.); 17393127867@163.com (K.Y.);; 2 Machinery Industry Pump Special Valve Engineering Research Center, Lanzhou 730050, China

**Keywords:** electric control valves (ECVs), fault diagnosis, variable working conditions, conditional adversarial learning, CEEMDAN

## Abstract

Electric control valves (ECVs) are core control components in process industries such as petrochemicals and power generation, and their operational reliability directly affects system safety and energy efficiency. However, frequent changes in the working conditions of ECVs cause vibration signals to exhibit strong nonlinearity and non-stationarity, which leads to the loss of high-frequency transient features, difficulty in extracting weak faults, and cross-condition domain shifts. These issues severely limit the generalization ability of existing fault diagnosis methods. To address this, this study proposes a collaborative fault diagnosis framework that combines a miniaturized high-frequency data acquisition system with a multi-scale attention-conditioned domain adversarial network (MS-ACDAN). First, a miniaturized high-speed data acquisition system is developed based on a field-programmable gate array (FPGA) to enable lossless acquisition of high-frequency transient signals. Subsequently, the original vibration signals are decomposed, filtered, and reconstructed using Adaptive Noise-Complete Empirical Mode Decomposition (CEEMDAN) and the Comprehensive Sensitivity Index (CSI) to generate feature-enhanced signals with high signal-to-noise ratios. Next, a feature extractor combining a one-dimensional convolutional neural network (1D-CNN) with a channel attention mechanism is constructed to automatically focus on key fault frequency band features while suppressing redundant information. Finally, a Conditional Adversarial Network (CDAN) is introduced for transfer learning. By establishing a conditional dependency between class prediction and feature representation. This approach achieves domain alignment while preserving the discriminative features of the data, thereby overcoming the limitation of traditional domain adaptation methods that ignore category information. The experimental results show that the proposed fault diagnosis framework demonstrates high recognition performance in various transfer tasks. Furthermore, even under extreme industrial noise conditions of 0 dB, the framework exhibits good diagnostic robustness. This research provides a theoretical basis and technical solution for addressing the fault diagnosis of critical control equipment under complex and variable working conditions.

## 1. Introduction

**Figure 1:**
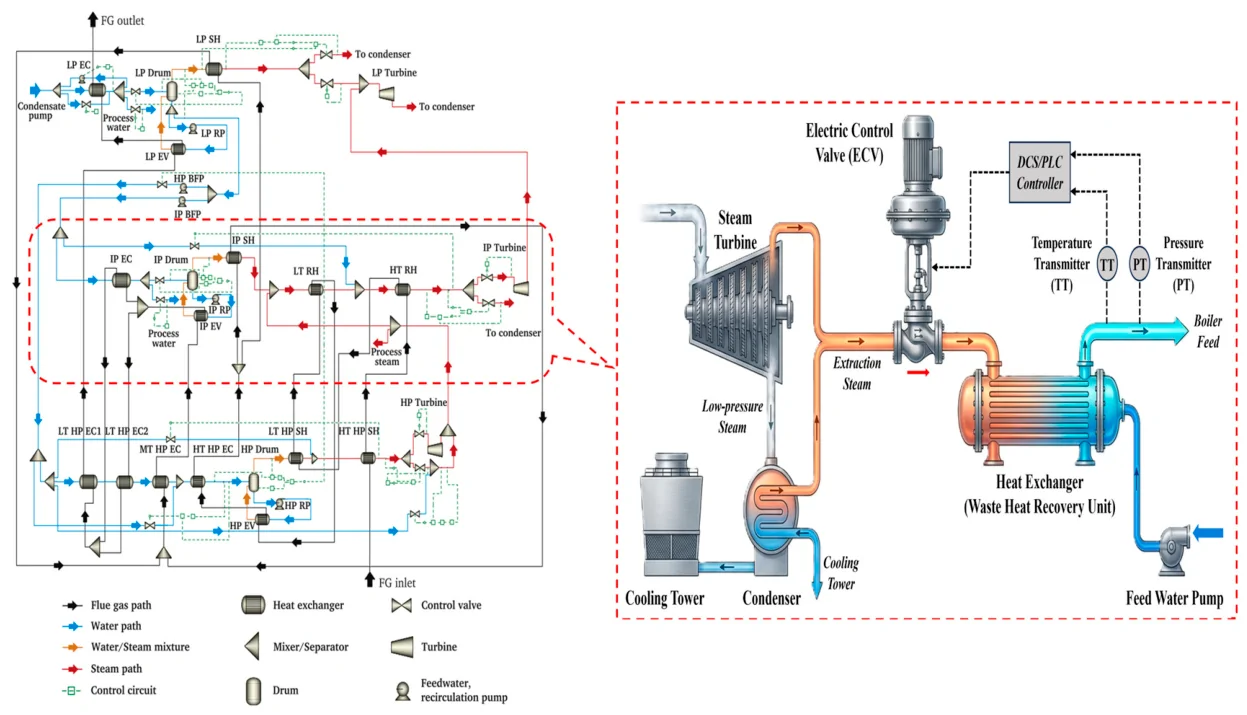
Schematic diagram of the power plant heat recovery system.

With global energy demand continuing to rise and the challenges of climate change becoming increasingly severe, improving energy efficiency and achieving energy savings in energy-intensive process industries such as petrochemicals, power generation, and metallurgy have become essential for sustainable development. These industries typically involve complex physical and chemical processes, where precise control of process parameters, including temperature, pressure, and flow rate, is closely related to production efficiency, product quality, energy consumption, and the overall safety and reliability of the system [1,2]. One of the critical devices for achieving this precise control is the ECV. With advances in automation technology, ECVs have been widely adopted in thermal systems for their high-precision control, fast response, and ease of integration with DCS/PLC systems. For example, they are used in steam turbine bypass systems to control thermal parameters, or deployed in heat exchanger loops to ensure efficient heat transfer [3,4]. Figure 1 illustrates a schematic of a waste heat recovery system in a power plant, where the ECV is a key control component. In actual industrial environments, ECVs are prone to various faults due to long-term exposure to harsh operating environments and frequent changes in working conditions, such as internal leakage, blockage, and packing leakage. Due to the complex fluid–structure interaction within the valve body, these early-stage failures typically manifest as strong nonlinear and non-stationary vibration signals. Without timely condition monitoring and accurate diagnosis, these failures can not only lead to energy waste, but may even result in catastrophic unplanned system shutdowns [5,6].

Reliable fault diagnosis depends not only on advanced algorithms, but also fundamentally on high-quality data acquisition [7]. Under variable working conditions, early valve faults typically generate weak vibration responses with low energy and broad frequency distributions. While ubiquitous in industry, traditional commercial data acquisition (DAQ) systems exhibit significant limitations when handling high-frequency, transient, and weak fluid excitation signals induced by condition variations. Existing commercial equipment is often bulky with closed hardware-software architectures, restricting the flexible deployment of sensing hardware at industrial edges in space-constrained sites. Moreover, these systems predominantly rely on USB or traditional serial ports for communication with host computers. Their limited bandwidth and operating system scheduling delays easily trigger data buffer overflows, leading to the random loss of crucial transient high-frequency fault features [8,9]. The distortion and aberration of this underlying data fundamentally limit the performance ceiling of subsequent diagnostic algorithms, causing the generalization ability of many advanced deep learning models to decline sharply after leaving the laboratory environment.

**Figure 2:**
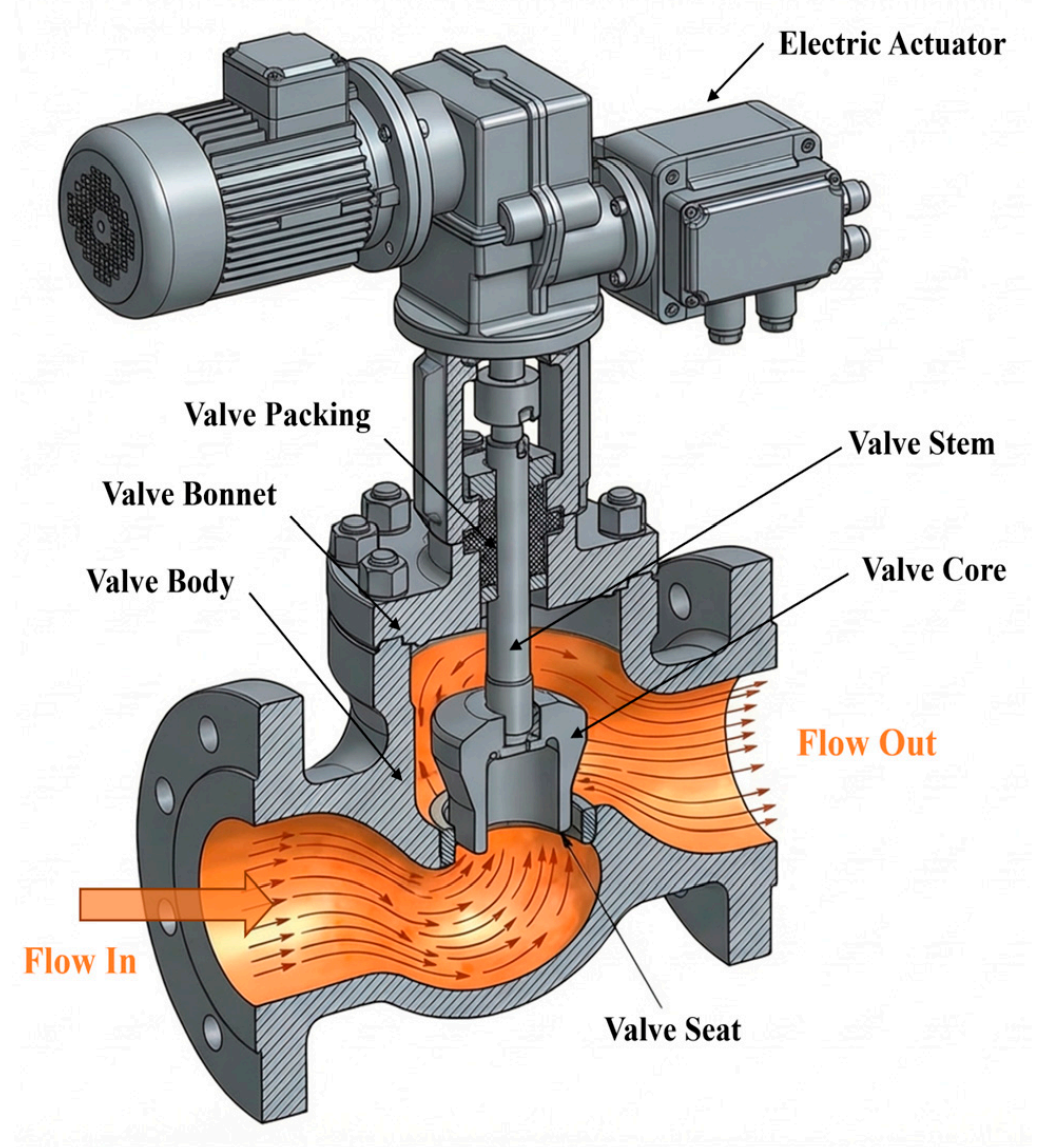
Structure of the electric control valve.

ECVs typically consist of an electric actuator, valve body, internal components (valve core and seat), valve stem, and valve packing, as shown in Figure 2. Even when high-quality raw signals are obtained, weak fault-related features under complex variable working conditions are often severely masked by strong background flow noise and industrial interference. Although traditional signal processing methods (such as Variational Mode Decomposition (VMD) and Empirical Mode Decomposition (EMD)) are widely used in data preprocessing, modal aliasing and endpoint effects remain inherent limitations in this process [10,11,12]. Adaptive noise ensemble empirical mode decomposition (CEEMDAN) performs well in suppressing non-stationary noise [13,14], but it ultimately only preprocesses the raw data, and subsequent fault feature extraction and discrimination still rely on manually designed features, which often require extensive expert experience. According to the development history of fault diagnosis methods, from early manual inspection, threshold alarms, and expert experience, to analytical models, to later shallow models such as support vector machines and neural networks, a data-driven transformation has been gradually realized. However, in actual variable working conditions (such as changes in pressure, temperature, flow, and valve opening), problems such as feature distribution drift, early weak feature signals being submerged by noise, and high overlap of multiple fault features lead to unsatisfactory fault diagnosis accuracy and robustness. Furthermore, excessive reliance on manual feature extraction has limited the ability of diagnostic models to extract deep, nonlinear fault feature information from raw signals [15,16,17,18,19]. In recent years, deep learning methods have achieved significant success in fault diagnosis due to their powerful, autonomous feature extraction capabilities [20,21]. However, in real industrial environments, the model’s diagnostic performance and generalization ability face severe challenges due to limitations in data acquisition costs, incomplete source-domain data, and variable working conditions [22]. For example, changes in ECV working conditions (such as upstream pressure fluctuations) can alter the statistical distribution of monitoring data, thus causing a “Domain Shift” phenomenon during fault diagnosis. This makes it difficult for models trained under fixed working conditions (Source Domain) to maintain their original diagnostic accuracy under new working conditions (Target Domain) [23].

Transfer learning and domain adaptation (DA) techniques are increasingly being introduced into the field of industrial fault diagnosis to mitigate the “domain shift” issues mentioned above. Adversarial domain adaptation methods have received widespread attention due to their end-to-end learning mechanisms [24,25,26]. The mainstream adversarial domain adaptation network frameworks mainly include: Adversarial Discriminative Domain Adaptation (ADDA), Gradient Reversal Layer (GRL)-based Domain Adversarial Neural Networks (DANN), and Wasserstein distance-based adversarial domain adaptation networks (such as WDGRL), etc. [27,28,29]. The above network models are good at extracting domain-invariant features and are committed to aligning the edge distribution of data, ignoring the alignment of class-conditional distributions. However, under varying working conditions, simply aligning the edge distribution may cause a misalignment of feature categories between the “source domain” and the “target” domain, resulting in negative transfer and reducing the accuracy of diagnosis and the generalization stability of the model [30]. Conditional Domain Adversarial Network (CDAN) integrates the prediction information of the classifier into the adversarial training, realizing the joint alignment of features and categories, which effectively addresses the issue of negative transfer [31,32]. Moreover, the feature extraction module of the standard CDAN framework, by treating all feature extraction channels fairly and equally, limits its ability to filter strong background noise (flow noise, mechanical noise, etc.) and prioritize fault-sensitive frequency bands, hindering class alignment under varying working conditions, thus posing a serious challenge to the generalization ability of the diagnostic model.

Considering the various challenges introduced by the aforementioned varying working conditions, we developed a miniaturized high-speed data acquisition system. We combined this system with a multi-scale attention-based conditional domain adversarial network (MS-ACDAN), creating a collaborative framework for fault diagnosis. The specific contributions of this paper are as follows:

(1) This paper independently developed a miniaturized high-frequency data acquisition system based on the FPGA. By leveraging the advantages of the FPGA’s underlying hardware in wideband sampling and lossless transmission, the system effectively avoids the packet loss and bandwidth limitations common in commercial data acquisition systems, thereby providing stable, high-quality data input for diagnostic models.

(2) A CEEMDAN-based multiscale decomposition strategy is proposed, combined with the Comprehensive Sensitivity Index (CSI) to filter and reconstruct features containing sensitive fault information, thereby achieving adaptive noise reduction and fault feature enhancement.

(3) An MS-ACDAN model with an integrated channel attention mechanism is constructed. It can not only adaptively focus on high signal-to-noise ratio fault bands, but also achieve domain alignment while retaining the discriminative features of the data by jointly aligning feature distribution and cross-domain features of categories, thereby improving diagnostic accuracy and generalization ability under different working conditions.

Comparative experiments on multiple transfer tasks show that the proposed method outperforms existing methods in both diagnostic performance and robustness.

The subsequent content of this paper is arranged as follows: Section 2 introduces the theoretical foundation and details of the MS-ACDAN framework; Section 3 elaborates on the FPGA-based data acquisition system, experimental platform construction, dataset construction, and evaluation metrics; Section 4 presents the results of comparative experiments, ablation experiments, and noise robustness evaluation; Section 5 summarizes the entire paper and looks forward to future work.

## 2. Theoretical Background

### 2.1. CEEMDAN Algorithm Overview

Empirical Mode Decomposition (EMD) is an adaptive time-frequency analysis method based on the signal’s own time scale. It is good at handling nonlinear and non-stationary signals. It can decompose several intrinsic mode functions (IMFs) without pre-setting basis functions, but it has the problem of mode aliasing [33]. To this end, the EEMD algorithm is proposed based on EMD. This algorithm eliminates white noise by adding white noise multiple times and ensemble averaging. However, the introduced white noise will affect the reconstruction accuracy [34]. This paper adopts noise-adaptive fully empirical mode decomposition (CEEMDAN). This method introduces noise adaptively, thereby more effectively suppressing mode aliasing and avoiding noise residue in the reconstructed signal [35]. Its algorithm decomposition process is as follows:

(1) First, the original vibration signal is decomposed by adding white noise using the EMD algorithm to obtain *IMF*_1,*i*_., and then averaged to obtain *IMF*_1_:

(1) $$F(n)=f(n)+\gamma_{0}\omega_{i}(n)$$

(2) $$IMF_{1}=\frac{1}{N}\sum_{i=1}^{N}IMF_{1,i}$$

where f(n) is the original signal; $\gamma_{0}$ is the amplitude of the added white noise; $\omega_{i}(n)$ is the amount of white noise added; *i* is the number of times the signal is constructed; and N is the average total number.

(2) The first-stage residual component $z_{1}(n)$ is calculated as

(3) $$z_{1}(n)=f(n)-IMF_{1}$$

(3) White noise $\gamma_{1}M_{1}(\omega_{i}(n))$ is added to the residual $z_{1}(n)$, and EMD is applied to obtain the second intrinsic mode function, *IMF*_2_:

(4) $$IMF_{2}=\frac{1}{N}\sum_{i=1}^{N}M_{1}(z_{1}(n)+\gamma_{1}M_{1}(\omega_{i}(n)))$$

In the formula, *IMF*_2_ is the modal component of the second stage; *M_j_* is set as the component of the j-th *IMF*_1_.

(4) This procedure is repeated for the (*j* + 1)-th stage, yielding the residual component $z_{j}(n)$ and the corresponding intrinsic mode function $MIF_{j+1}$:

(5) $$z_{j}(n)=z_{j-1}(n)-MIF_{j}$$

(6) $$IMF_{j+1}(n)=\frac{1}{N}\sum_{i=1}^{N}MIF_{1}(z_{1}(n)+\gamma_{j}MIF_{j}(\omega_{i}(n)))$$

(5) The above steps are repeated until the residual signal can no longer be decomposed by EMD. The final CEEMDAN result consists of multiple intrinsic mode functions and a residual component, expressed as

(7) $$F(n)=\sum_{j=1}^{J}IMF_{j}+z_{j}(n)$$

where *J* represents the total number of modes in the modal decomposition process.

Compared to traditional EMD and EEMD, the most obvious improvement of CEEMDAN is that it can effectively alleviate modal aliasing while maintaining better spectral separation, without accumulating residual noise during the reconstruction process, and signal integrity is preserved. After CEEMDAN of the original signal, the original vibration signal can be decomposed into multi-scale characteristic components, and then those sensitive components related to faults can be selected, and components dominated by noise can be suppressed.

### 2.2. Conditional Domain Adversarial Network

The core objective of domain adaptation (DA) is to reduce the distribution gap between data in the source domain and the target domain. Taking the classic domain adversarial neural network (DANN) as an example, it primarily relies on adversarial training to bring the marginal distributions of the two domains closer together, thereby extracting domain-invariant features. However, as working conditions change, the degree of feature shift varies across different fault categories. Simply aligning the edge distribution can easily confuse the boundaries of the domain categories, ultimately leading to a decline in diagnostic performance [36,37].

The Conditional Domain Adversarial Network (CDAN) introduces a multilinear conditioning mechanism into the adversarial learning framework, facilitating the joint distribution alignment of feature representations (*f*) and classifier predictions (*g*). Let the feature vector output by the deep feature extractor be denoted as $f\in {\mathbb{R}}^{d}$, and the class probability vector output by the classification network as $g\in {\mathbb{R}}^{c}$ (where c represents the number of categories). Rather than feeding f directly into the domain discriminator, CDAN constructs a multilinear mapping combination (outer product) of *f* and *g*, denoted as T(f,g)=f⊗g. This joint representation serves as the input to the conditional domain discriminator, *D*. Through a minimax game training process, CDAN forces the domain discriminator to become incapable of distinguishing whether the joint features originate from the source or target domain. This mechanism strictly preserves category discriminability during the feature alignment process.

## 3. Proposed MS-ACDAN Methodology

To address the challenges of extracting weak fault features and the low cross-domain recognition accuracy in ECVs under variable working conditions, this section details the proposed MS-ACDAN framework. This architecture comprises three core modules: signal enhancement, a multi-scale feature extractor integrated with a channel attention mechanism, and conditional domain adversarial training.

### 3.1. Signal Enhancement via CSI-Based IMF Selection

The vibration signals of ECVs originate from mechanical impacts, fluid-induced pulsations, and multi-physical coupling responses arising from fluid–structure interaction. This coupling mechanism inherently produces nonlinear and non-stationary characteristics in fault-related signals. Furthermore, feature drift among different fault categories under variable working conditions further increases diagnostic complexity. From a physical perspective, multi-scale signal processing is required to effectively separate fault-related components from background noise.

After CEEMDAN, the original vibration signal is separated into multiple intrinsic mode functions (IMFs). However, these components may contain spurious modes and redundant flow-induced noise. To accurately identify sensitive components containing genuine fault excitation information, a Comprehensive Sensitivity Index (CSI) is proposed based on kurtosis (K), correlation coefficient (CC), and energy ratio (ER). By normalizing and integrating these three indicators, the CSI avoids contradictory evaluations that may arise from relying on a single metric and provides a more comprehensive assessment of signal quality. The CSI is defined as

(8) $$CSI=\alpha \cdot \hat{K_{j}}+\beta \cdot \hat{CC_{j}}+\gamma \cdot \hat{ER_{j}}$$

where $\hat{K_{j}}$, $\hat{CC_{j}}$, and $\hat{ER_{j}}$ represent the normalized kurtosis, correlation coefficient, and energy ratio of the k-th IMF, respectively. The coefficients α, β and γ denote weighting factors. Through repeated experimental validation, it is found that setting α=0.4, β=γ=0.3 effectively suppresses interference components while enhancing fault-sensitive features. Finally, the CSI values for all the IMFs are calculated, and their mean is established as an adaptive threshold. IMFs with a CSI value greater than or equal to this threshold are selected and reconstructed to obtain the enhanced signal. This reconstructed sequence effectively filters out background fluid interference while maximally preserving transient fault dynamics, serving as the input for the subsequent deep learning network.

### 3.2. Multi-Scale Attention Feature Extractor

Different fault types of ECVs, such as high-frequency jet excitation associated with internal leakage and low-frequency pulsation caused by obstruction, exhibit distinct frequency-band characteristics. To enhance the network’s capability to capture multi-scale features and suppress residual noise during feature extraction, a multi-scale attention-based feature extractor is designed.

The module is built upon a one-dimensional convolutional neural network (1D-CNN) architecture. Convolution kernels with different receptive field sizes are applied in parallel to capture rich time–frequency representations, including local high-frequency details and global low-frequency trends [38,39]. The outputs of the parallel convolutional branches are fused to form a multi-scale feature representation.

To automatically adjust the importance of different feature channels, a channel attention mechanism is incorporated. Given a fused feature map $U\in {\mathbb{R}}^{L\times C_{in}}$, where *L* denotes sequence length, and *C* denotes the number of channels, global average pooling (GAP) is first applied to compress the feature map into a one-dimensional channel descriptor $z\in {\mathbb{R}}^{1\times C_{in}}$:

(9) $$z_{c}=\frac{1}{L}\sum_{i=1}^{L}U_{c}(i)$$

Subsequently, two fully connected layers are employed to model nonlinear interdependencies among channels, and a *Sigmoid* activation function is applied to generate a normalized attention weight vector $W\in {\mathbb{R}}^{1\times C_{in}}$. Ultimately, the weight vector is multiplied element-wise with the original feature map *U* along the channel dimension, generating the recalibrated feature map $U^{\prime }$:

(10) $$U_{c}^{\prime }=W_{c}\cdot U_{c}$$

This attention mechanism enables the network to adaptively emphasize fault-sensitive feature channels while suppressing channels dominated by working condition variations or background noise. Consequently, more discriminative feature representations are provided for cross-domain adaptation.

### 3.3. The Overall Framework of MS-ACDAN

**Figure 3:**
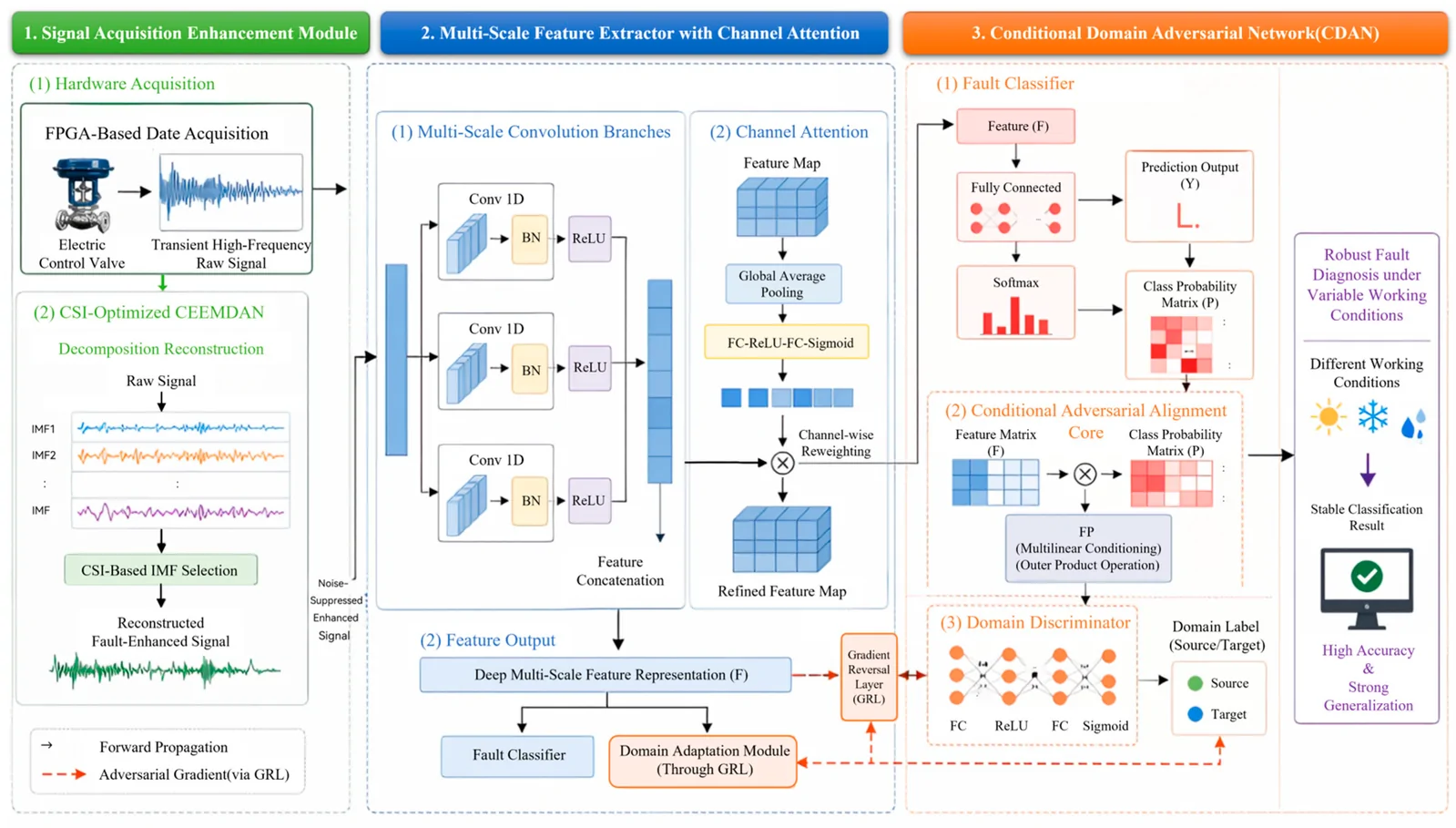
Overall architecture and diagnostic process of the MS-ACDAN framework.

The proposed MS-ACDAN framework is developed based on unsupervised domain adaptation within a deep transfer learning paradigm [40,41]. It integrates signal enhancement, a multi-scale attention feature extractor, and a conditional adversarial learning module to improve diagnostic performance in the target domain. Detailed parameters of the framework are listed in Table 1, while its architectural structure and diagnostic workflow are depicted in Figure 3.

The cross-domain fault diagnosis task involves a labeled source-domain dataset $D_{s}={\left\{\left(x_{i}^{s},y_{i}^{s}\right)\right\}}_{i=1}^{n_{s}}$ and an unlabeled target-domain dataset $D_{t}={\left\{\left(x_{j}^{t}\right)\right\}}_{j=1}^{n_{t}}$. The overall network consists of a feature extractor $G_{f}$, a state classifier $G_{c}$, and a conditional domain discriminator $G_{d}$.

The network training process alternates between supervised learning and adversarial adaptation. In the source domain supervised learning branch, the feature extractor and classifier compute the cross-entropy loss based on source domain labels:

(11) $$l_{cls}(G_{f},G_{c})=-\frac{1}{n_{s}}\sum_{i=1}^{n_{s}}L_{CE}(G_{c}(G_{f}(x_{i}^{s})),y_{i}^{s})$$

In the domain adaptation branch, the feature vector $f=G_{f}(x)$ from both domains and the soft label prediction $g=G_{f}(x)$ undergo an outer product operation to form the conditional feature vector h=f⊗g. The conditional domain discriminator, $G_{d}$, attempts to accurately distinguish whether the input *h* originates from the source or target domain. Conversely, the feature extractor, $G_{f}$, maximizes the classification error of the domain discriminator via a Gradient Reversal Layer (GRL). The domain adversarial loss is defined as

(12) $$\begin{array}{l} l_{adv}(G_{f},G_{c},G_{d})=-\frac{1}{n_{s}}\sum_{i=1}^{n_{s}}\log(G_{d}(f_{i}^{s}⊗g_{i}^{s}))\\ -\frac{1}{n_{t}}\sum_{t=1}^{n_{t}}\log(1-G_{d}(f_{j}^{t}⊗g_{j}^{t})) \end{array}$$

The overall optimization objective of MS-ACDAN forms a minimax game, expressed as

(13) $$\min_{G_{f},G_{c}}\max_{G_{d}}l_{total}=l_{cls}-\lambda l_{adv}$$

where λ is a penalty coefficient balancing classification and domain alignment objectives. Through this adversarial training process, MS-ACDAN achieves joint alignment of both marginal and class-conditional distributions in the feature space. As a result, even when domain shifts arise due to varying working conditions, the model maintains clear decision boundaries and produces accurate diagnostic outputs in the target domain.

## 4. Experimental Setup and Data Acquisition System

All the models are trained and tested on the same dataset, with a fixed random seed of 42 to ensure the reproducibility of the experimental results. Considering potential fluctuations caused by randomness, each experiment is repeated five times, and the final results are reported as the mean ± standard deviation. We used Windows 11 and the PyTorch 2.5.1 framework, with computations accelerated by an NVIDIA RTX 4050 laptop GPU (6 GB). For hyperparameters, we employed a greedy search combined with empirically determined hyperparameter values [42]. Training used the Adam optimizer (initial learning rate set to 5 × 10^−4^, weight decay set to 1 × 10^−4^) combined with a cosine-annealed learning rate scheduler (minimum learning rate set to 1 × 10^−6^), running for a total of 200 epochs with a batch size of 32. The specific configurations of each network layer are shown in Table 1.

### 4.1. FPGA-Based Data Acquisition System and Experimental Platform

In practical applications, early-stage, weak faults in ECVs under varying working conditions (such as slight internal leaks) often exhibit low signal energy and a wide frequency band, making them highly susceptible to high-frequency transient interference, such as fluid noise. Traditional commercial data acquisition systems typically suffer from large hardware footprints and closed hardware architectures that prevent secondary development, limiting their adaptability in compact industrial monitoring scenarios. Moreover, these devices rely on general-purpose buses and PC operating systems for communication scheduling, frequently encountering bandwidth limitations and latency issues when processing massive amounts of high-frequency continuous vibration data. This can lead to hardware buffer overflows and the loss of transient, weak fault characteristics. It should be clarified that the FPGA system is responsible for real-time data acquisition. The CEEMDAN preprocessing and MS-ACDAN inference are executed on a host PC, and no embedded inference is performed on the FPGA platform.

**Figure 4:**
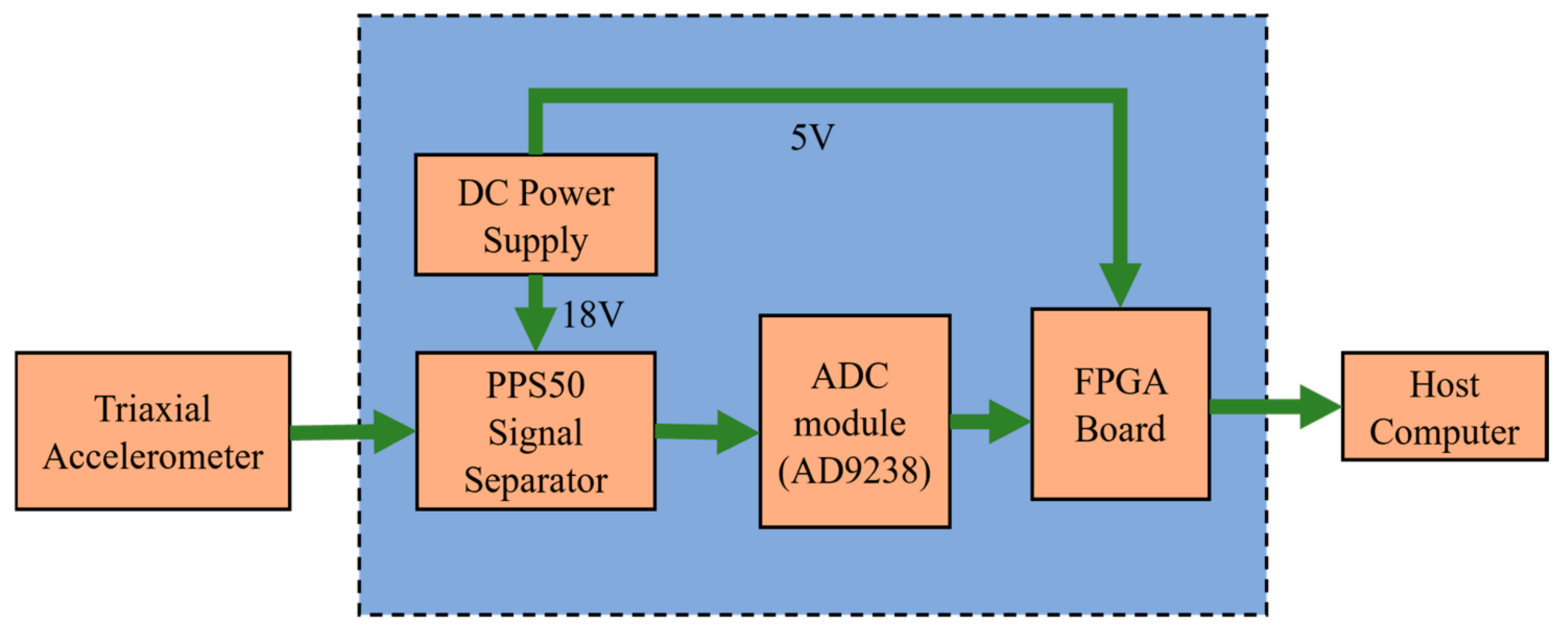
The hardware architecture of the vibration data acquisition system.

To address the aforementioned shortcomings, we designed a miniaturized high-speed data acquisition system based on the FPGA, specifically for simultaneously capturing weak low-frequency and transient high-frequency fault vibration signals under varying working conditions. In terms of hardware, we selected a high-sampling-rate FPGA development board and an AD conversion module as the critical hardware components to ensure effective acquisition of vibration signals [43,44]. The hardware architecture of the entire system is shown in Figure 4.

**Figure 5:**
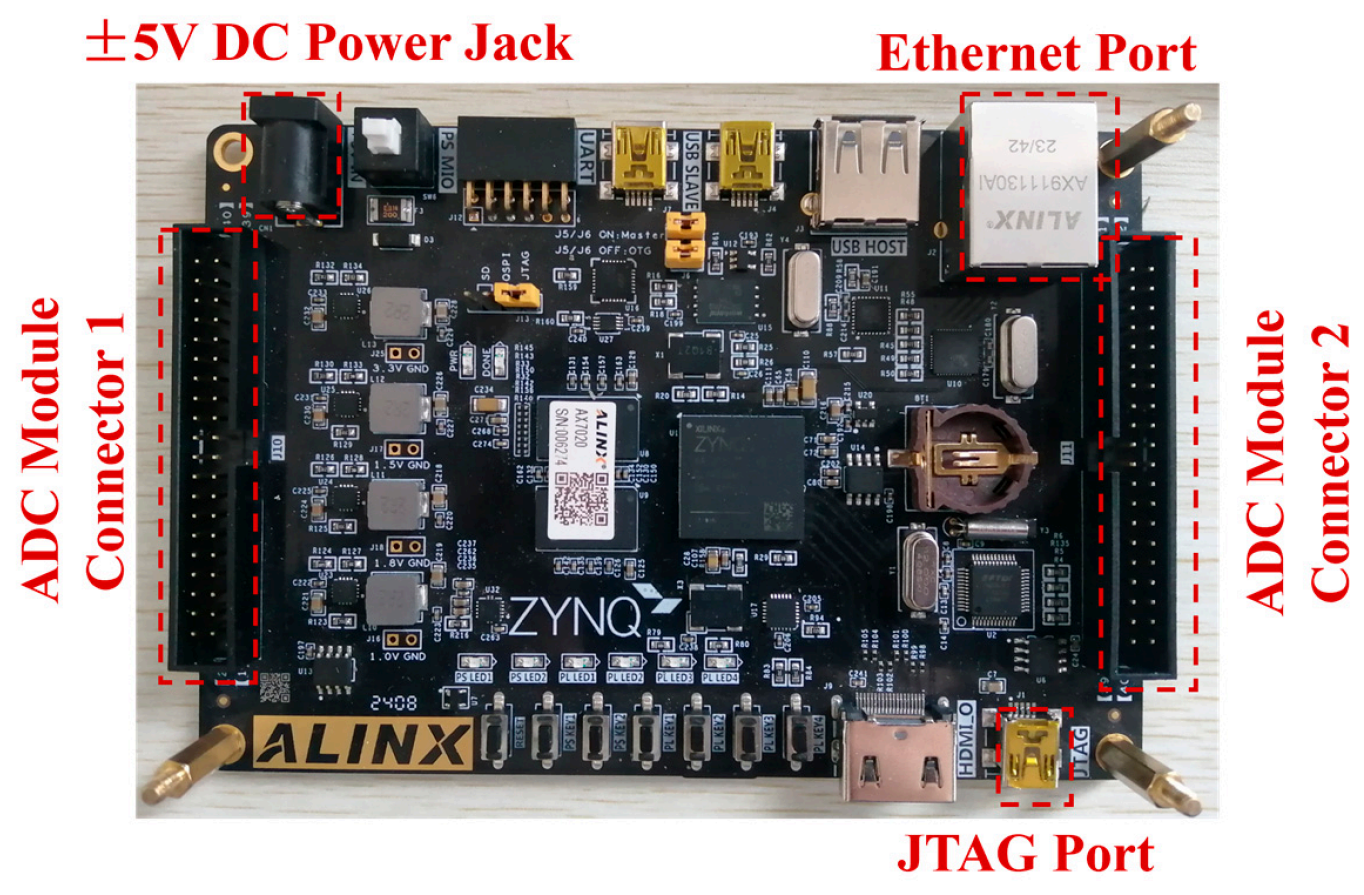
The FPGA development board.

The FPGA development board used in the system is the Zynq-7000 series XC7Z020-1CLG400I development board, as shown in Figure 5. This development board integrates a high-speed, industrial-grade AXI bus interface between the FPGA and the ARM, which facilitates the internal transmission of acquired data within the board. Additionally, the board has a large-capacity high-speed DDR3 chip for data caching and operating system operation, and provides serial and Ethernet communication interfaces for data transmission with external devices. The AD converter module used is the AD9238 module, which supports multiple conversion channels, 12-bit sampling data, and an analog signal input range of 5 V to +5 V. This module has a standard 2.54 mm pitch 40-pin header for connection to the FPGA development board, and the analog signal input interface is an SMA interface.

**Figure 6:**
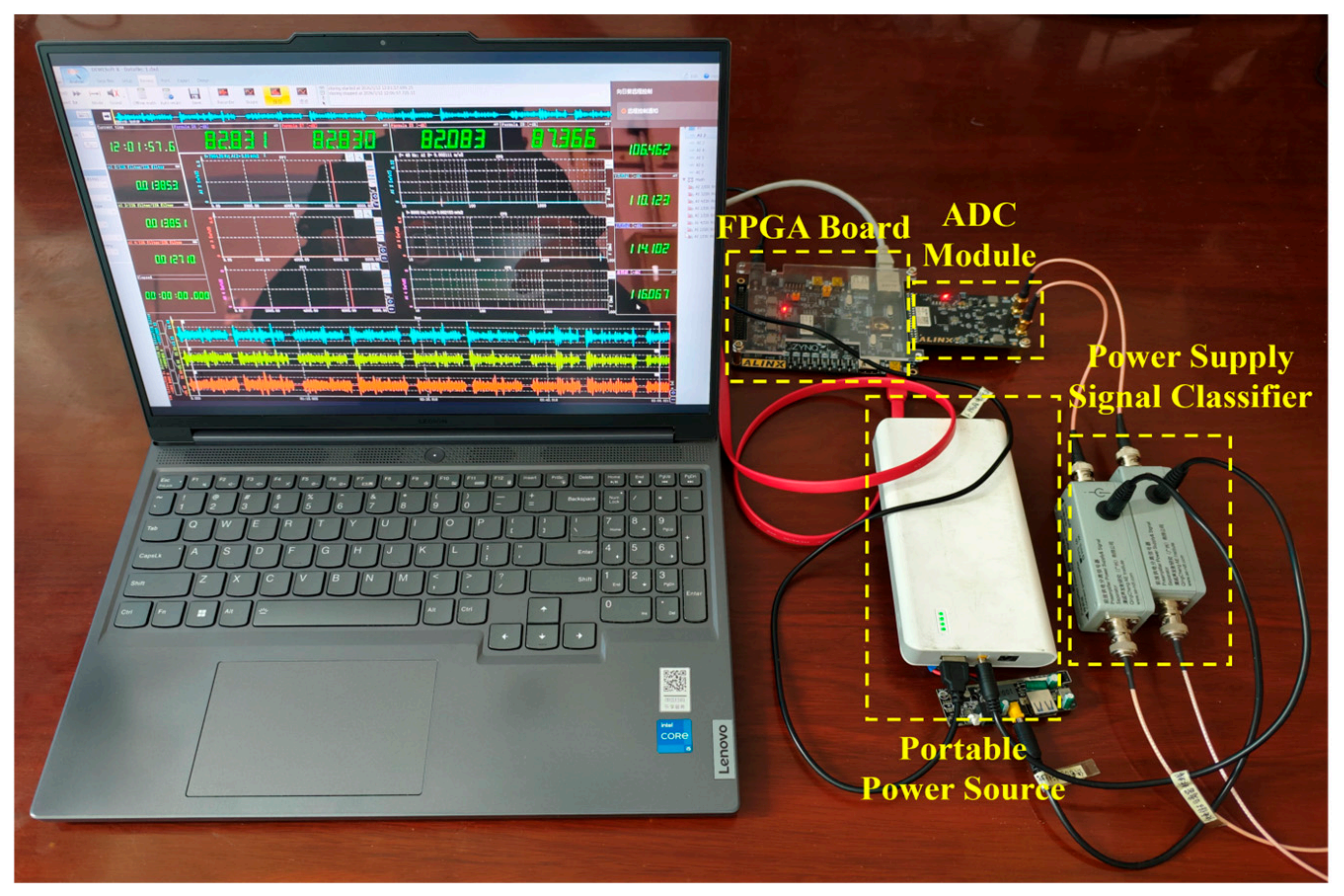
FPGA-based high-speed vibration data acquisition system.

Figure 6 shows the diagram of the miniaturized FPGA high-frequency acquisition system. The hardware of this system includes a tri-axial accelerometer, power supply signal classifier splitter, ADC module, FPGA development board, low-noise cables, power supply cables, Ethernet cable, host computer, and portable power source. The vibration signal is acquired using a tri-axial accelerometer with a sampling frequency range of 1 Hz to 16 kHz, a resonance frequency of 40 kHz, a sensitivity of 100 mV/g, and an output voltage range of −5 V to +5 V. The triaxial accelerometer converts the acquired vibration signals into voltage signals, which are transmitted to the AD9238 digital-to-analog converter module via the low-noise cable. This module supports multiple conversion channels, 12-bit sampling data bits, and an analog signal input range of −5 V to +5 V. Digital circuit programming is performed on the FPGA development board to drive the AD9238 ADC board for fault signal data acquisition and voltage-to-digital signal conversion. The obtained digital signals are transmitted to the FPGA development board for data processing and buffering. The buffered data is then uploaded to the host computer via Ethernet for data processing and analysis. The triaxial accelerometer is powered by a matching PPS50 power supply signal classifier, and 18 V and 5 V voltages are provided to the power supply signal classifier and FPGA development board, respectively, through a mobile power supply.

Compared with conventional centralized DAQ systems, the FPGA-based compact high-speed data acquisition system offers improved real-time performance, enhanced buffering stability, and reduced risk of data loss during long-term monitoring. The introduction of this system eliminates transient feature loss under variable working conditions at the physical layer, ensuring a high signal-to-noise ratio and feature fidelity. This provides a reliable data foundation for validating the cross-domain generalization capability of the proposed MS-ACDAN framework.

**Figure 7:**
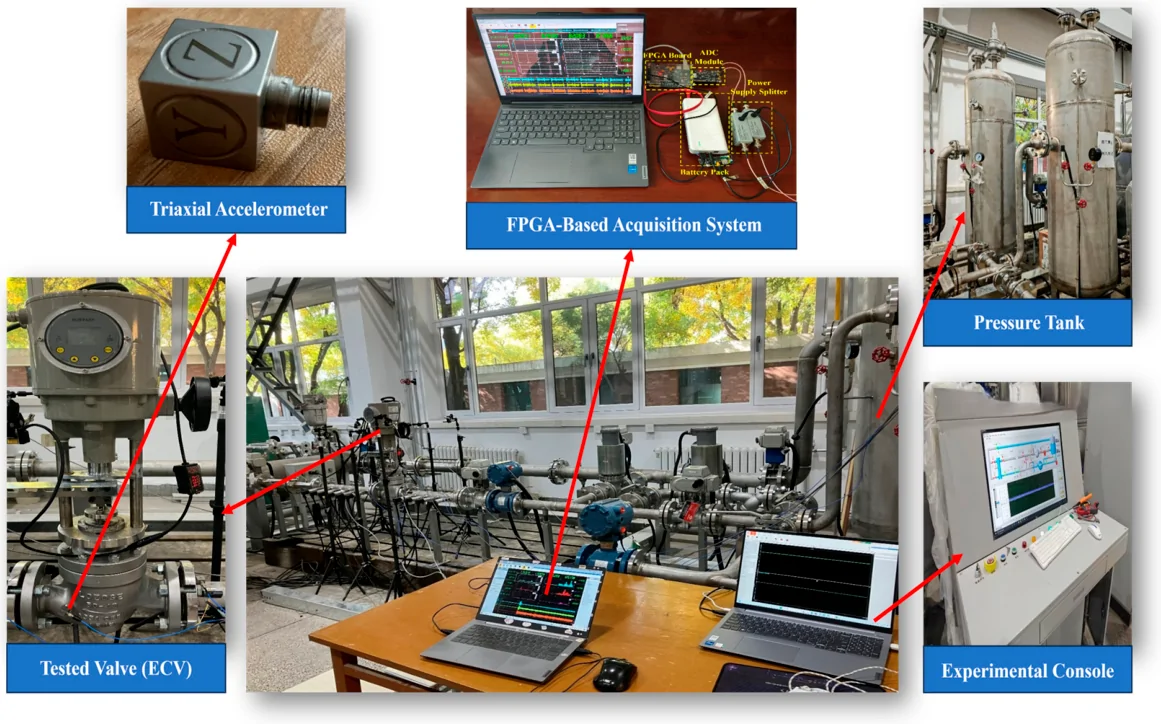
Electric control valve fault simulation experimental platform.

**Figure 8:**
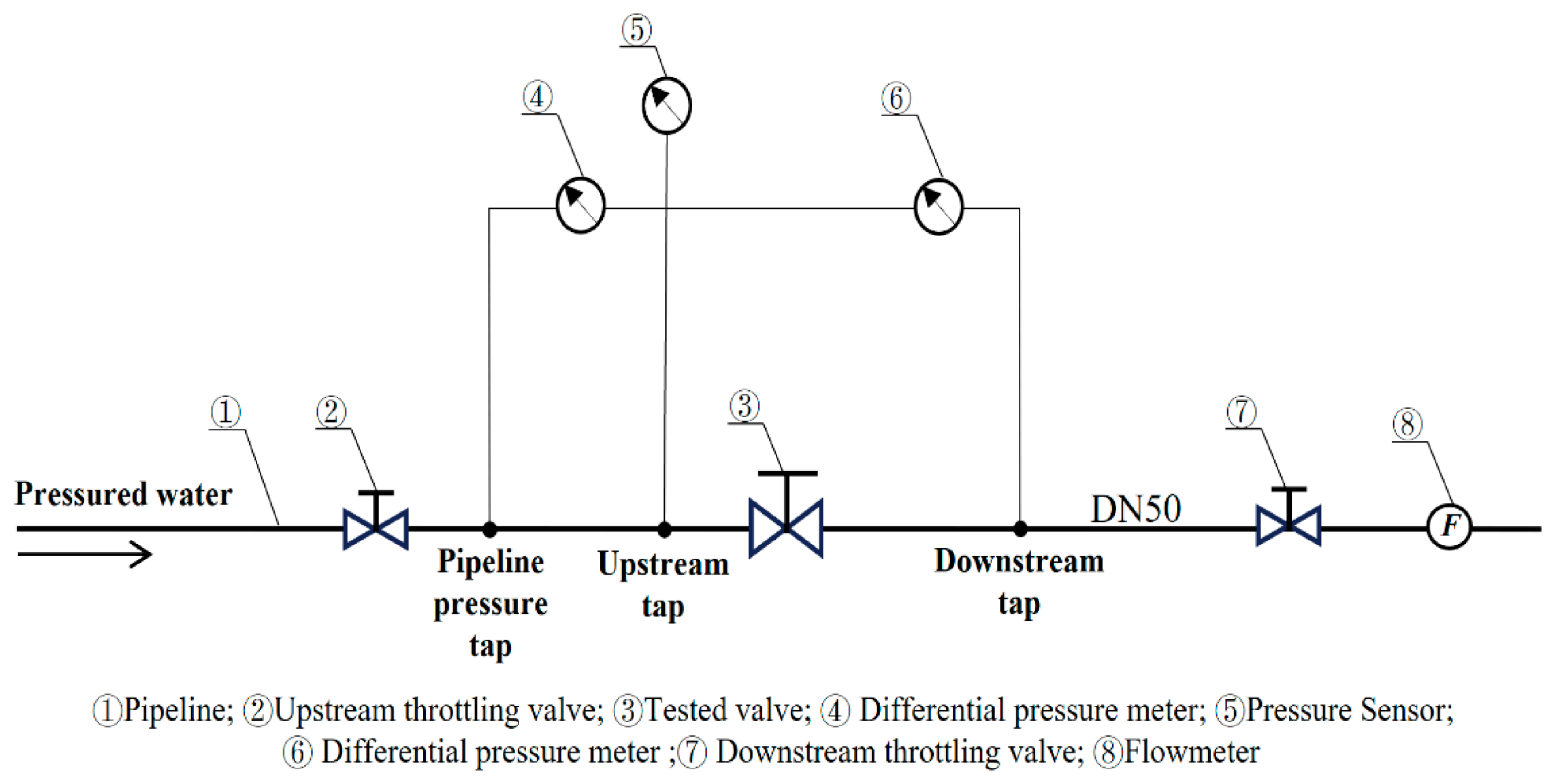
Experimental system working principle diagram.

In practical applications, continuous monitoring of ECVs across all working conditions in a power plant heat recovery system is difficult. Therefore, a fault simulation experimental platform is designed and constructed to emulate three typical fault modes under three different working conditions, as shown in Figure 7. The system mainly consists of the tested valve, data acquisition system, pressure stabilization tank, and experimental control console. A PN40 DN50 single-seat ECV is selected as the test object, and the working principle of the system is illustrated in Figure 8. A tri-axial accelerometer is used to measure vibration signals along the X, Y, and Z directions. To minimize signal attenuation and maintain experimental consistency, the *Z*-axis vibration signal is selected for subsequent analysis.

To evaluate the generalization ability of MS-ACDAN under variable working conditions and its capability to mitigate domain shift, three working conditions are established by adjusting the upstream pressure. Under each working condition, vibration signals corresponding to the four predefined states are acquired. The detailed experimental scheme is presented in Table 2.

To rigorously evaluate the generalization ability of the proposed MS-ACDAN framework under different working conditions, we constructed three cross-domain transfer tasks using datasets collected from three different working conditions (labeled A, B, and C, respectively). The specific settings of the source and target domains are shown in Table 3.

### 4.2. Equivalent Degradation State Definition

To systematically evaluate the diagnostic performance of the proposed MS-ACDAN framework under variable working conditions, multiple working states are simulated on the constructed experimental platform. It must be noted that creating actual physical defects (e.g., real plug erosion) through mechanical machining or electrochemical corrosion inside in-service valves within high-pressure fluid systems is irreversible, costly, and poses extreme safety risks, including pipe bursts or loss of control [15]. Therefore, based on the principles of fluid dynamic equivalence and vibration–acoustic homology, a “state equivalent simulation” strategy is adopted to scientifically replicate typical ECV fault characteristics.

**Figure 9:**
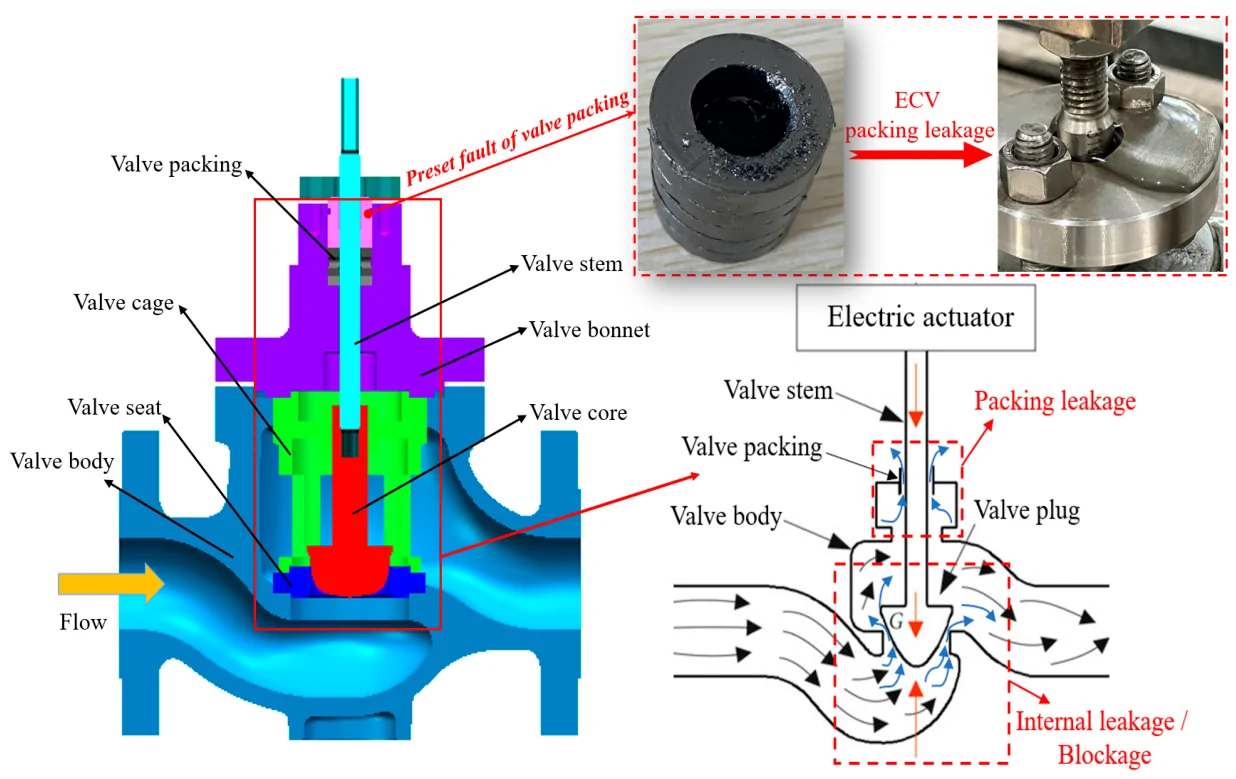
Schematic diagram of preset faults for internal leakage, partial blockage, and packing leakage in ECV.

The purpose is not to replicate the exact physical form of actual faults, but rather to reproduce the equivalent dynamic vibration response characteristics caused by typical valve degradation under controlled, repeatable laboratory conditions. Specifically, preset faults are introduced by precisely controlling the opening degree of ECVs, constructing four representative preset fault states, as shown in Figure 9. The preset faults are defined as follows:

**(1) Normal State:** The valve remains fully closed, the sealing surfaces are tightly seated, and there is no leakage.

**(2) Internal Leakage (equivalent 3% opening):** The valve opening is precisely locked at 3%. This state simulates valve internal leakage caused by poor sealing contact due to corrosion or wear of the valve core through the use of a micro-flow channel (slight internal leakage). This micro-flow channel can reproduce the flow disturbances and vibration characteristics caused by early-stage deterioration of sealing performance or slight internal leakage.

**(3) Partial Blockage (equivalent 5% opening):** This is achieved by adjusting the valve opening to 5%. This state simulates an equivalent resistance-fluctuation type fault caused by reduced flow area due to deposits and partial blockage of the internal flow channel. This definition can reproduce the local blockage vibration response associated with disturbed flow patterns and local turbulence.

**(4) Packing Leakage (Physical Damage):** Introduce actual damage to the packing seal through controlled mechanical wear to replicate packing leakage faults caused by packing aging or fatigue.

Based on the aforementioned pre-defined faults, the proposed pre-defined fault method, while ensuring repeatability, controllability, and safety, can also reproduce the flow distortion and unsteady vibration response characteristics observed in actual industrial scenarios. Furthermore, this equivalent simulated fault state has been widely used in laboratory fault diagnosis research to establish benchmark datasets under controlled conditions, thereby providing reliable data support for verifying the diagnostic performance and generalization ability of the algorithm.

### 4.3. Dataset Description and Preprocessing

In this study, a triaxial accelerometer is employed to acquire vibration signals from the ECV under three working conditions and four states (normal and three fault states). The sampling frequency is 16 kHz, and each acquisition lasted 8 s. To increase the dataset while preserving local temporal dynamic characteristics, a resampling strategy is adopted. The sliding window length is 1024, and the step size is 512 (50% overlap). Before applying the sliding window, the data is strictly segmented based on working conditions (domains) to prevent data leaks. This ensures that windowing and feature extraction are performed independently on the source and target data. This strategy generated 996 small samples for each working condition, totaling 2988 samples across the three conditions (249 samples per category for each working condition, as detailed in Table 4). Subsequently, all the resampled small samples are used to construct a multi-scale information fusion dataset (IFD) using a CEEMDAN-based multi-scale decomposition strategy combined with the Comprehensive Sensitivity Index (CSI). The constructed IFD integrates information across different scales, enabling a more comprehensive characterization of the ECV’s operating state and providing a robust data foundation for subsequent fault diagnosis, which is used for training and evaluating fault diagnosis models under varying operating conditions.

**Figure 10:**
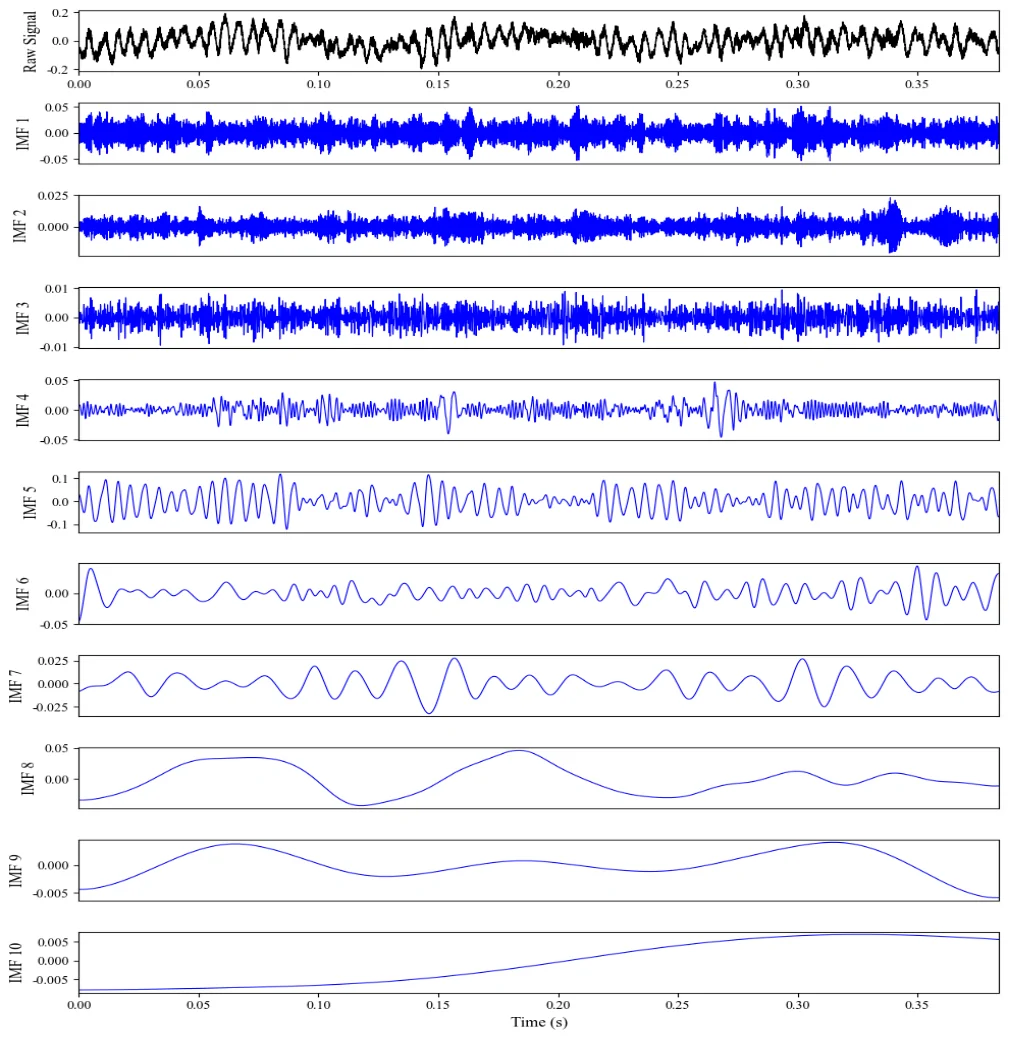
CEEMDAN results.

Given the computational complexity of the CEEMDAN algorithm and its requirements for signal stability, a segment of length *N* = 6144 consecutive sampling points (corresponding to *T* = 0.384 s) is extracted from the stable middle part of the original signal for visualization analysis. This length is sufficient to ensure that the envelope spectrum analysis has the necessary frequency resolution. Taking the internal leakage fault signal of the ECV under 1.5 MPa pressure as an example, its CEEMDAN result is shown in Figure 10. The signal is decomposed into 10 intrinsic mode functions (IMFs) from high frequency to low frequency. It can be seen that the original signal exhibits significant nonlinear and non-stationary characteristics. The noise component is mainly concentrated in earlier IMFs (such as IMF4–IMF6), while certain specific fluid pulsation or background noise features may be hidden in the IMFs.

**Figure 11:**
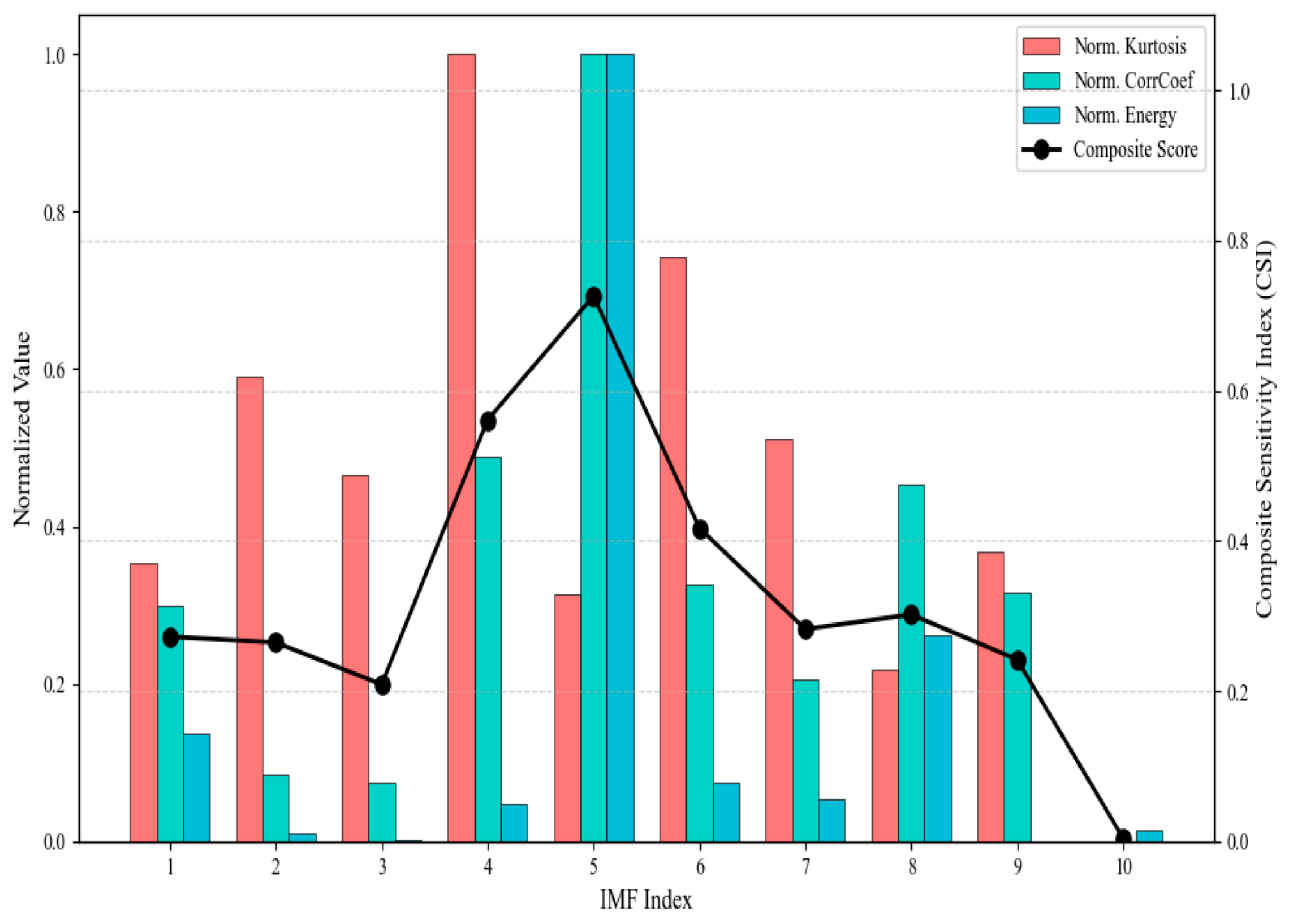
Multi-parameter selection criteria for IMFs.

Figure 11 illustrates the IMF reconstruction process based on the Comprehensive Sensitivity Index (CSI). Several IMFs exhibit high kurtosis and energy ratios, indicating that they contain rich fault information. The proposed CSI metric, using the average CSI value (0.3279) as the selection threshold, effectively integrates these features. The “black line” in the figure visually displays the optimal IMF components—IMF4, IMF5, and IMF6—with a total of three optimal components selected.

**Figure 12:**
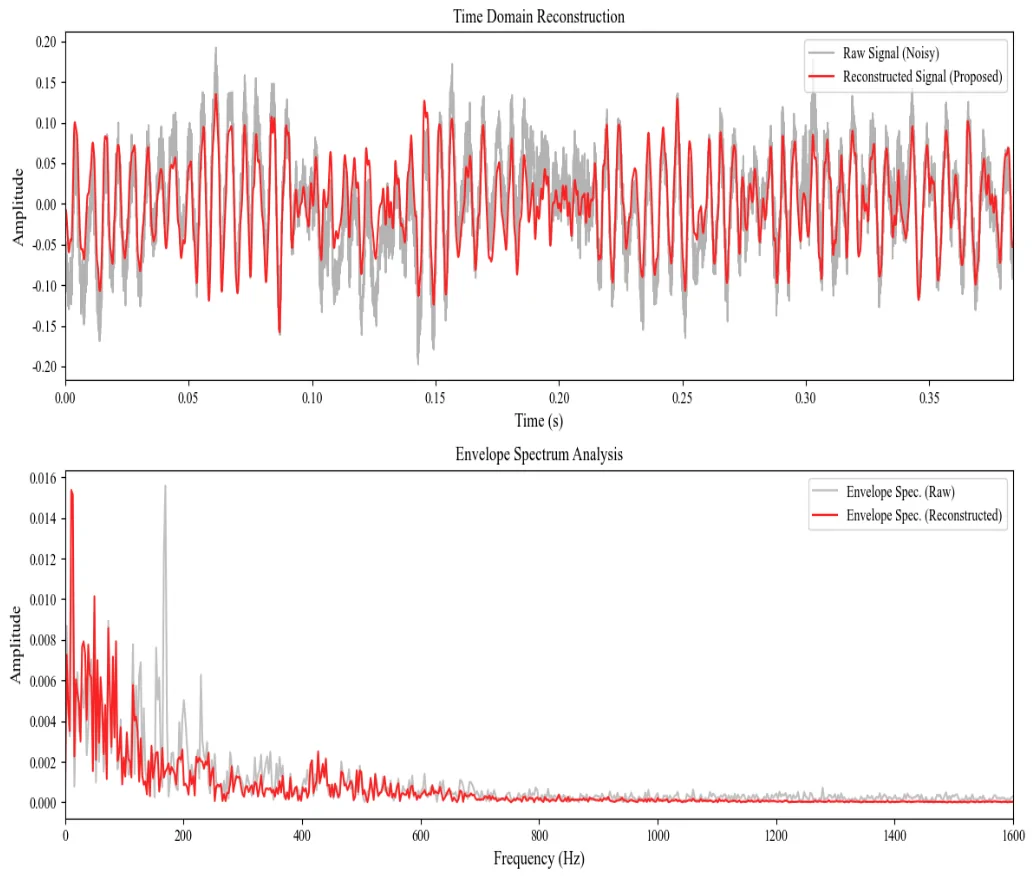
Time-domain and envelope-spectrum comparisons between original and reconstructed signals.

Figure 12 shows a time-frequency domain comparison of the original and reconstructed signals. In the time-domain waveform (top panel), the background noise in the reconstructed signal (red line) is significantly attenuated compared to the original signal (gray line), indicating that the CEEMDAN-CSI-based multi-scale reconstruction effectively suppresses background noise. The envelope spectral analysis (bottom panel) further shows that the frequency-domain energy of the original signal is primarily concentrated at 180 Hz, where a significant interference peak is observed. This frequency component is more conservatively interpreted as a non-fault-related excitation, such as mechanical vibration of an upstream water pump or resonant response of a local structure. This strong interference is effectively suppressed using the proposed reconstruction method. In contrast, a low-frequency component near 20 Hz becomes clearly distinguishable in the reconstructed spectrum. Although the precise physical origin of this frequency band requires dedicated fluid–structure interaction analysis, it is consistently observable under the internal leakage condition in the present dataset. The significant enhancement of this stable 20 Hz component confirms that the proposed strategy successfully extracts flow-excited weak fault characteristics from background noise. In addition, we emphasize that Figure 12 serves as a representative example to illustrate the effectiveness of the proposed CEEMDAN-CSI preprocessing strategy in enhancing weak fault-related components. A comprehensive inter-class and cross-condition discrimination analysis is presented in Section 5 through confusion matrices and feature distribution visualizations.

### 4.4. Evaluation Metrics

To rigorously evaluate the fault diagnosis performance and generalization ability of the proposed method under varying working conditions, this paper introduces a multi-dimensional evaluation metric. These metrics are *Accuracy*, *Precision*, *Recall*, and *F*_1_-*score*. The specific calculation formulas are as follows:

(14) $$Acc=\frac{T_{P}+T_{N}}{T_{P}+T_{N}+F_{P}+F_{N}}$$

(15) $$P=\frac{T_{P}}{T_{P}+F_{P}}$$

(16) $$R=\frac{T_{P}}{T_{P}+F_{N}}$$

(17) $$F_{1}=2\times \frac{P\times R}{P+R}$$

where *T_P_* (*True Positives*) represents the number of positive samples correctly predicted by the model; *T_N_* (*True Negatives*) represents the number of negative samples correctly predicted; *F_P_* (*False Positives*) represents the number of negative samples incorrectly predicted as positive; and *F_N_* (*False Negatives*) represents the number of positive samples incorrectly predicted as negative.

This fault diagnosis task is a multi-class classification problem. To reduce the impact of class distribution imbalance on the evaluation results, we adopted the macro-averaging strategy to calculate *Precision*, *Recall*, and *F*_1_-*score* [45]. This strategy first calculates the above metrics for each class separately, then computes the arithmetic mean by assigning equal weights to each class. Thus, the final evaluation metrics can objectively measure the model’s ability to identify various types of faults.

To visualize the MS-ACDAN framework’s ability to handle domain translation problems under varying working conditions, we employ confusion matrices and the t-SNE algorithm as visual evaluation tools [46,47]. The confusion matrix illustrates the distribution of misclassifications among categories in the target domain test set. t-SNE projects high-dimensional features onto a two-dimensional plane, clearly showing cross-domain feature alignment and category clustering boundaries. To ensure rigorous reproducibility and prevent the generation of artificial clusters driven by random hyperparameter selection, the t-SNE algorithm is explicitly implemented using Principal Component Analysis (PCA) initialization, a perplexity parameter set to 30, a specific learning rate of 200, and a maximum limit of 1000 iterations. The random seed is set to 42, consistent with the training configuration, to ensure stable and reproducible embeddings across repeated runs. It should be noted that t-SNE is employed solely as a qualitative visualization tool rather than a quantitative evaluation metric.

## 5. Experimental Validation and Result Analysis

### 5.1. Comparison with Representative Baseline Models

**Figure 13:**
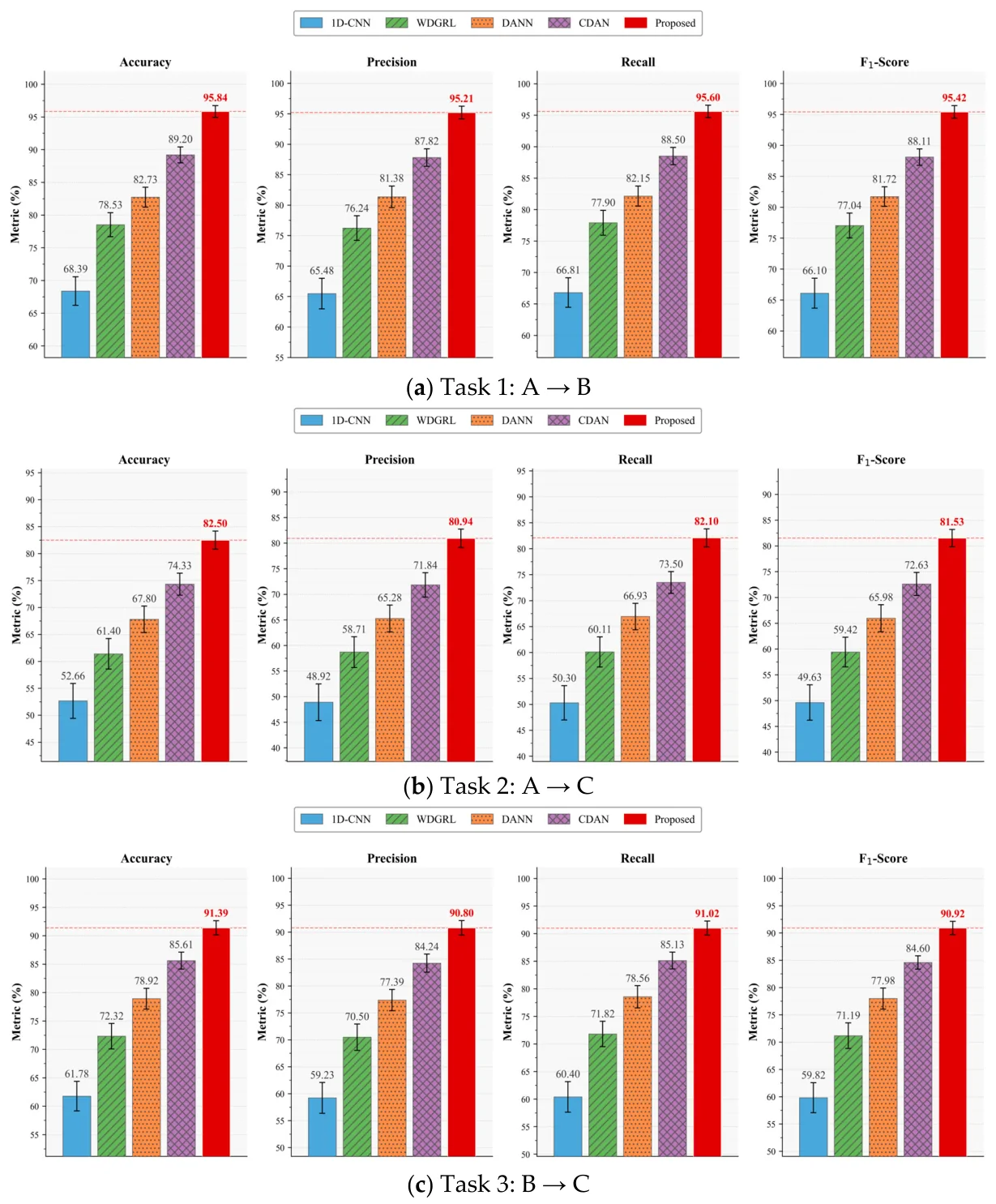
Evaluation metrics for cross-work condition generalization experiments.

To comprehensively evaluate the effectiveness and superiority of the proposed method under varying working conditions, three sets of cross-condition diagnostic tasks are established based on the evaluation scheme described in Section 4.1. Four representative fault diagnosis frameworks are selected for comparative experiments: one-dimensional convolutional neural networks (1D-CNN), domain adversarial neural networks (DANN), WDGRL networks, and standard conditional domain adversarial networks (CDAN). The evaluation results are shown in Figure 13. To ensure fairness, all the compared models are trained using the same hyperparameter settings and the same preprocessing strategy.

As shown in Figure 13, the MS-ACDAN model proposed in this paper outperforms other comparison models in all three transfer tasks, achieving an average fault diagnosis accuracy of 90.0%. In particular, the highest recognition accuracy is achieved in Task 1, which reached 95.84%. However, in the most challenging Task 2, its accuracy reached 82.50%, which is 8.2% higher than that of the standard CDAN. This significant improvement results from the fact that the proposed method not only achieves precise joint category alignment but also enhances the signal-to-noise ratio of weak transient features through high-quality data acquisition systems and multiscale data preprocessing strategies. In contrast, in Task 2 with the largest pressure variations, the average accuracy of the one-dimensional convolutional neural network (1D-CNN) is only 52.66%. This result indicates that the feature distributions are severely domain-shifted due to drastic changes in working conditions, thereby considerably reducing the model’s generalization ability. Although the transfer learning methods DANN and WDGRL introduced marginal distribution alignment, their generalization ability still notably declined, with average accuracies dropping to 67.80% and 61.40%, respectively. These findings show that simply aligning marginal distributions while ignoring the alignment of conditional distributions causes misalignment of features near the decision boundary, thereby reducing diagnostic performance. By contrast, the standard CDAN model addresses cross-domain misclassification by jointly aligning marginal and conditional distributions, achieving an accuracy of 74.33%, which significantly outperforms the approaches that rely on marginal alignment alone.

**Figure 14:**
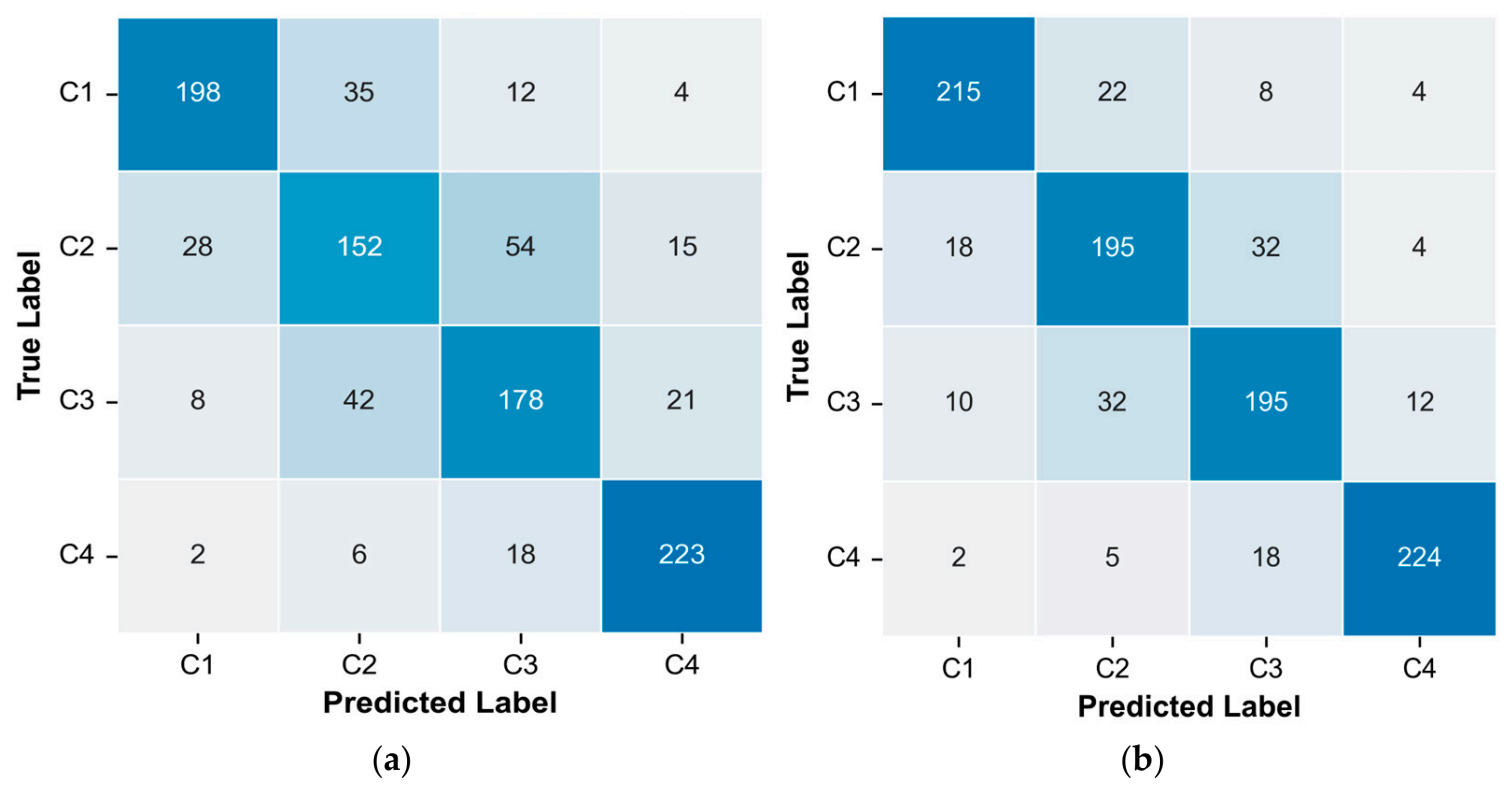
Confusion matrices of standard CDAN (**a**) and MS-ACDAN (**b**) on Task 2.

To more intuitively compare the feature mapping performance of different models under extreme working condition changes, we selected *Task 2* (1 MPa → 2 MPa), which exhibits the most drastic working condition changes, for visualization analysis. In this paper, we use confusion matrices and t-SNE to illustrate the distribution in the feature space. As shown in Figure 14, the standard CDAN model exhibits severe confusion between C1 and C2 under high-pressure target conditions. This is because the increase in upstream pressure significantly amplifies flow noise, thereby masking the weak high-frequency features associated with early-stage valve internal leakage. Conversely, the MS-ACDAN model performs relatively better, markedly reducing misclassifications between C1 and C2 as well as between C1 and C3. Its accuracy improved from 75.4% to 83.2%, indicating a significant enhancement in discriminative capability.

**Figure 15:**
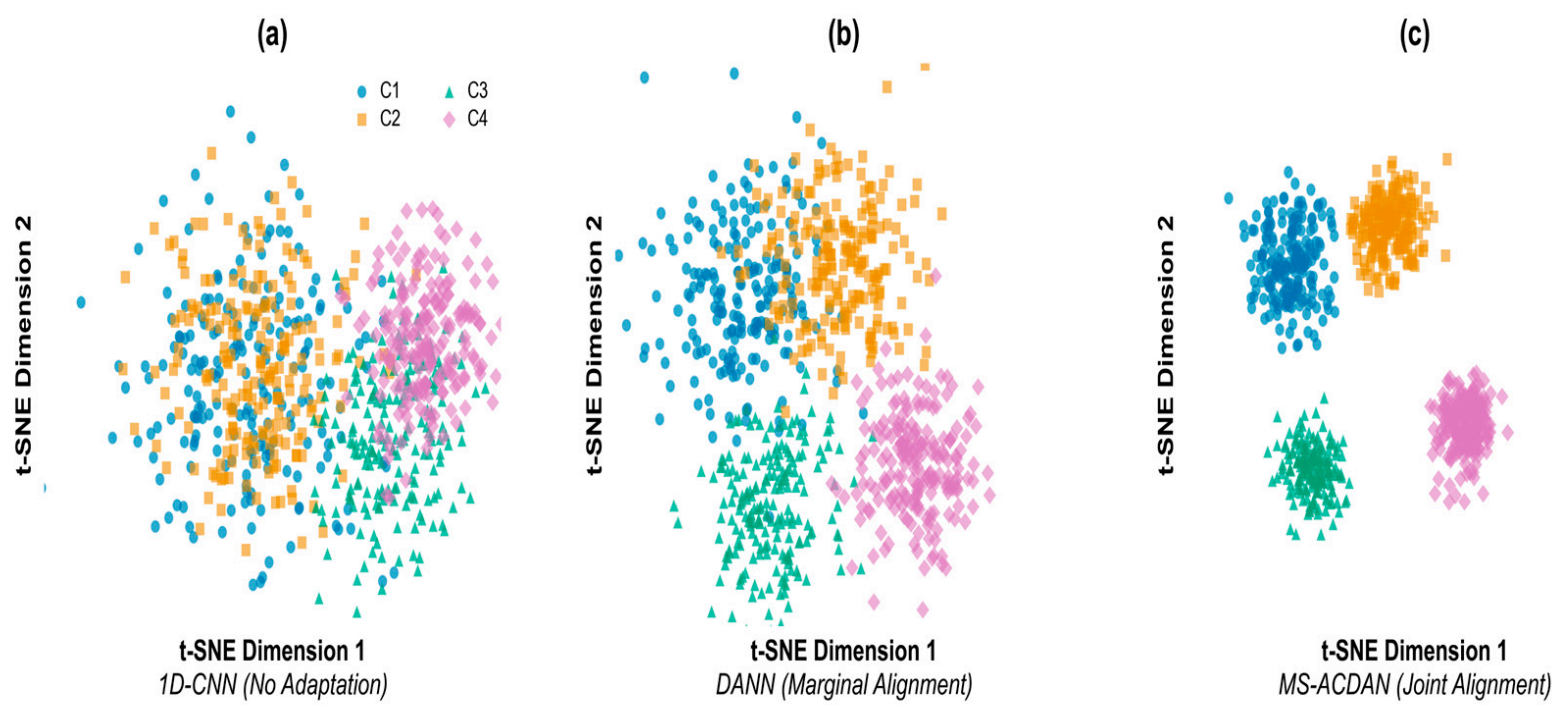
t-SNE visualization of learned features on Task 2. (**a**) 1D-CNN (no adaptation) exhibits severe class overlap. (**b**) DANN (marginal alignment) reduces overlap but maintains large intra-class scatter. (**c**) MS-ACDAN (joint alignment) achieves compact intra-class clusters and clear inter-class boundaries.

The t-SNE visualization in *Task 2* clearly illustrates the alignment effect of cross-domain feature distributions. Figure 15 clearly shows the evolution from feature entanglement (1D-CNN) to tight clustering (MS-ACDAN). Figure 15a, the 1D-CNN without domain adaptation exhibits not only severe feature entanglement but also an overall disorganized distribution; in Figure 15b, after margin alignment, the DANN can roughly separate the classes, but there is still significant overlap between C1 (blue circle) and C2 (orange square); in Figure 15c, MS-ACDAN shows tightly clustered intra-class features and relatively wide inter-class boundaries, improving both domain invariance and discriminative ability. Overall, MS-ACDAN effectively filters out redundant information caused by fluid disturbances and maintains class separation under varying working conditions, demonstrating the feasibility of our proposed multi-scale attention-based conditional adversarial framework in addressing domain shift.

### 5.2. Ablation Analysis of MS-ACDAN Components

**Figure 16:**
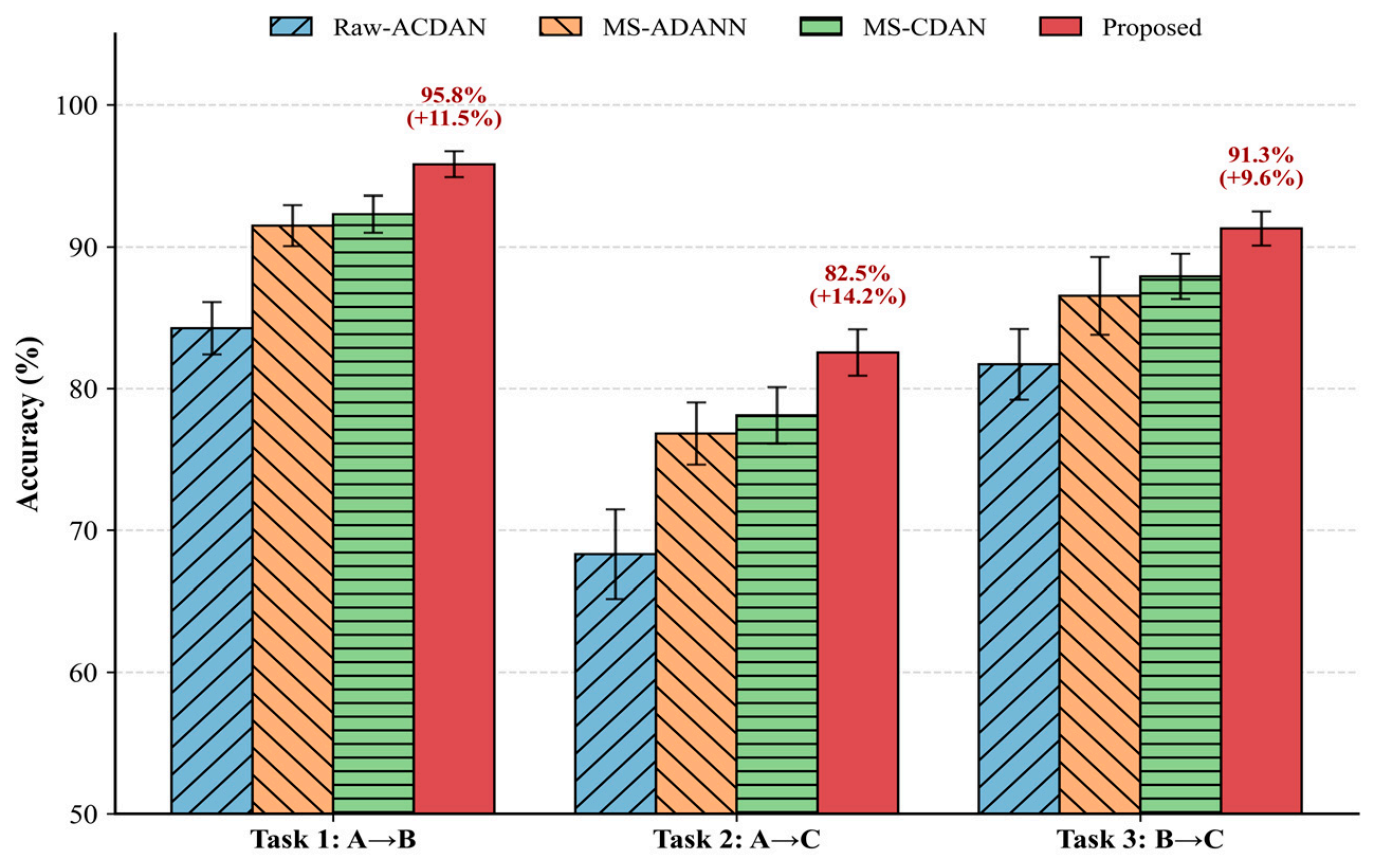
Accuracy comparison of MS-ACDAN and its ablative variants on three cross-domain tasks.

To verify the contributions of each module in the MS-ACDAN framework, we conducted ablation experiments on the proposed method. The experimental results are summarized in Table 5 and Figure 16. We designed three network variants for the ablation experiments:

(1) Raw-ACDAN: Remove the CSI-based CEEMDAN data preprocessing module and feed the raw signals acquired by the FPGA directly into the feature extractor.

(2) MS-CDAN: Remove the channel attention mechanism in the feature extractor and replace it with the standard 1D-CNN multi-scale convolutional layer with equal weights.

(3) MS-ADANN: Replace the conditional domain adversarial in MS-ACDAN with the standard DANN, which means that only the marginal distribution is aligned, while the conditional distribution is ignored.

Table 5 shows that the complete model outperforms all three variant models, and removing any module affects accuracy. Specifically, removing the data preprocessing module, i.e., the CSI-based CEEMDAN signal enhancement module, reduces the accuracy of the three tasks by 11.6%, 12.2%, and 8.6%, respectively, which is the largest decrease among the three variants. This confirms that data preprocessing is the cornerstone of the entire process and makes the greatest contribution. For MS-CDAN, which removes the channel attention mechanism, accuracy decreased by 3.5% to 4.9%, indicating that the attention mechanism effectively helps the model filter out fault-sensitive frequency bands while suppressing irrelevant features, thereby enhancing feature discriminability; For the MS-ADANN model, which replaces the CDAN module with the DANN, the performance drop is the fastest in Task 2, with accuracy falling to 76.82%, the largest overall performance decrease (4.3–5.7%). Clearly, this is because the DANN only aligns the edge distribution and does not consider the class-conditional distribution of the classifier in the target domain, while the CDAN incorporates class-conditional information into the alignment process, naturally better meeting the actual needs of classification tasks. Consequently, the complete MS-ACDAN outperformed all the evaluation metrics, indicating that signal enhancement, attention feature extraction, and joint distribution alignment together form a complementary triad, which is supported by both theoretical and experimental analyses.

The bar chart in Figure 16 compares the accuracy of the three variants and the complete model across three transfer tasks. The height of each bar represents the average of five independent experiments, and the error bars reflect the standard deviation. The red numbers above the “Proposed” bar chart indicate the improvement compared to the Raw-ACDAN model. As can be seen from the figure, the Raw-ACDAN model without the signal enhancement module performs the worst, which demonstrates the necessity of executing data preprocessing operations before domain alignment. *Task 2* (1 MPa → 2 MPa) showed the largest variation in working conditions, with all the variants generally exhibiting low accuracy and being most sensitive to module removal. This indicates that the more severe the domain shift, the greater the difficulty in feature extraction and domain adaptation. In contrast, the complete MS-ACDAN model achieved the highest accuracy and lowest standard deviation across the three tasks, validating the effectiveness and rationality of our proposed integrated architecture.

### 5.3. Noise Robustness Evaluation

**Figure 17:**
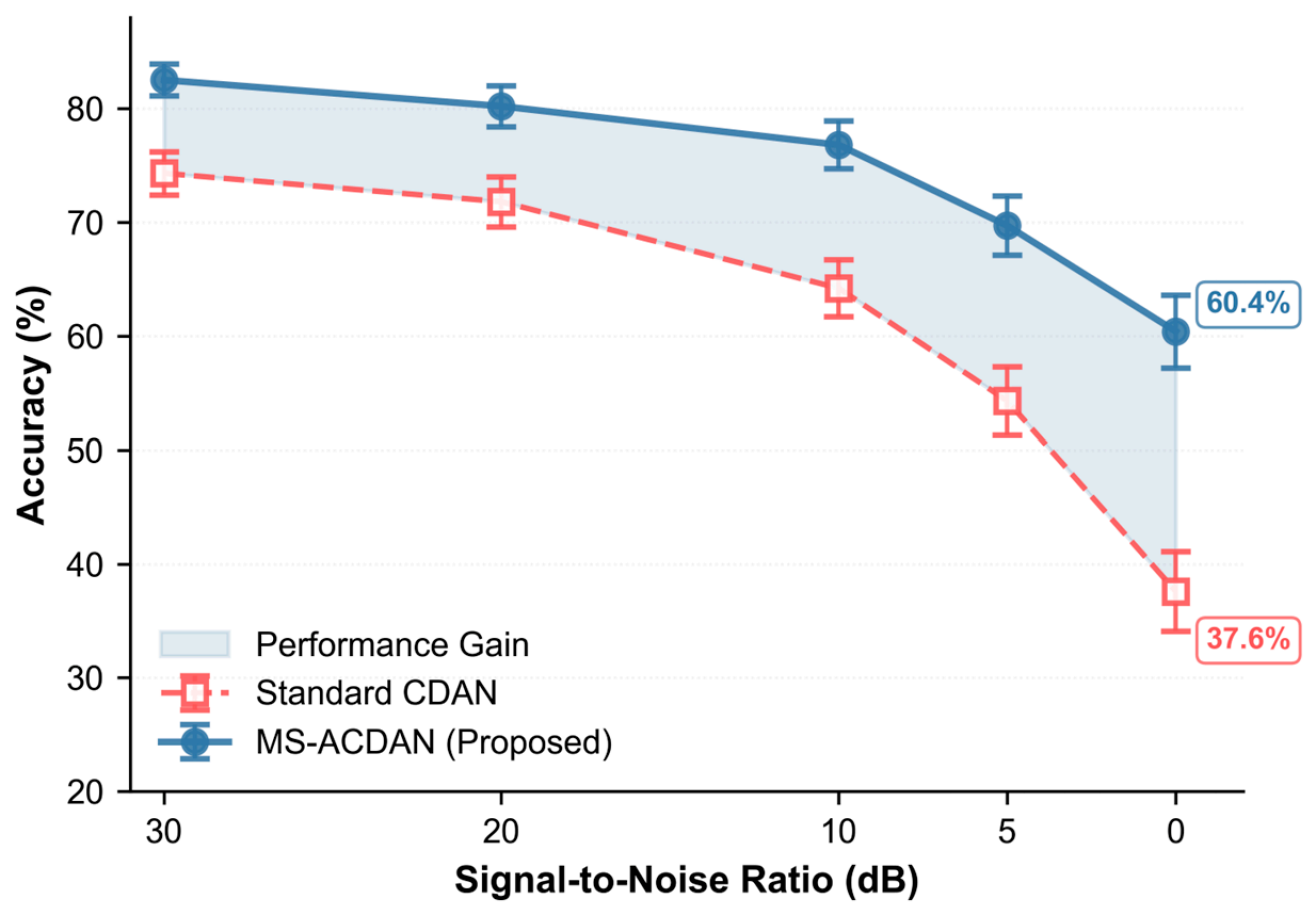
Accuracy comparison of MS-ACDAN and Standard CDAN under varying SNR levels on Task 2.

To test the performance of the proposed method in noisy environments, we selected *Task 2* (1 MPa → 2 MPa), which exhibits the most significant changes in working conditions. Different intensities of Gaussian white noise are injected into the target domain signal, with signal-to-noise ratios (SNRs) set to 30, 20, 10, 5, and 0 dB, respectively. The introduced Gaussian white noise covered conditions ranging from near-clean laboratory environments to extremely harsh industrial noise scenarios [45,46]. Figure 17 shows the accuracy trends of MS-ACDAN and the standard CDAN under different SNRs using a line graph with standard deviation error bars.

Figure 17 displays the diagnostic accuracy of the standard CDAN and MS-ACDAN at different signal-to-noise ratios (SNRs), where the error bars represent the standard deviation from five independent trials. As the SNR decreases from 30 dB to 0 dB, the accuracy of both models declines; however, MS-ACDAN outperforms CDAN at all noise levels, with the performance gap between the two gradually widening from 8.2% at 30 dB to 22.8% at 0 dB. Even under extreme noise conditions (0 dB), MS-ACDAN maintains an accuracy of 60.4%. The shaded area in Figure 17 also visually highlights the performance improvement achieved by MS-ACDAN. Furthermore, MS-ACDAN’s accuracy decay rate of 0.74%/dB is nearly 40% (39.3%) slower than CDAN’s decay rate of 1.22%/dB, indicating better stability. Taking a comprehensive view, MS-ACDAN’s noise-resistant performance advantages derive from the combined effects of high-quality front-end data acquisition, CSI-based CEEMDAN data preprocessing, and multiscale feature learning, enabling it to maintain reliable diagnostic performance and robustness even in harsh industrial environments.

## 6. Conclusions

To address the critical challenges associated with weak fault features, pronounced nonlinear and non-stationary signal characteristics, and domain shift-induced generalization degradation under variable working conditions of ECVs, this study developed a multi-scale attention-based diagnostic framework integrating signal enhancement with conditional domain adversarial transfer learning. Unlike conventional approaches that rely solely on data-driven post-processing algorithms, the present work incorporates a self-developed FPGA-based high-frequency data acquisition system to eliminate packet loss and distortion of transient flow-field features at the physical acquisition stage. The results demonstrate that CEEMDAN combined with CSI-based adaptive selection effectively reconstructs high signal-to-noise ratio signals. When integrated with multi-scale convolution and channel attention mechanisms to enhance fault-sensitive frequency representations, and further combined with conditional domain adversarial learning for class-conditional distribution alignment, the proposed framework significantly improves cross-condition diagnostic accuracy and stability. Across multiple transfer tasks and noisy environments, the proposed method consistently outperforms baseline models and exhibits reduced performance degradation.

This paper addresses the limitation of traditional domain adaptive methods, which only align edge distributions and ignore class-based conditional distributions by first enhancing the signal, then extracting multi-scale discriminative features, and finally aligning conditional class distributions. Furthermore, the introduction of multi-scale feature extraction and channel attention mechanisms makes the model more sensitive to weak fault features under complex fluid disturbances. In engineering terms, the FPGA-based high-frequency acquisition system ensures signal integrity and real-time performance, providing a reliable data foundation for capturing the dynamic changes in early-stage faults. The experimental results indicate that the proposed method achieved an average accuracy of 90.0% across three transfer tasks and maintained good stability under various noise environments. These findings provide a feasible approach for condition monitoring and predictive maintenance of critical control equipment in complex process industries.

In summary, while this study achieved promising results under experimental conditions, it still has some limitations. First, the experimental data are derived from simulated fault states, while the fault state simulation strategy faces certain physical limitations; future work will require using long-term in-field operational degradation states as fault states and utilizing these data for further validation. Second, the current study only addresses single-source-domain adaptation; future work will be able to explore extending this to multi-source-domain or continuously varying working condition scenarios. Third, for real-time embedded deployment requirements, the model needs to reduce computational complexity while maintaining high diagnostic accuracy. Fourth, Future research may also explore advanced multi-task fusion frameworks such as mixture-of-experts architectures, simulated data-assisted diagnosis, and partial transfer adaptation methods for improved robustness under incomplete source data or extreme domain shifts. Therefore, future work will focus on industrial field data verification, continuous variable working condition modeling, and lightweight architecture design to improve the engineering applicability and scalability of the proposed method.

## Figures and Tables

**Table 1:** Detailed architecture and parameters of the MS-ACDAN.

No.	Module	Type	Input Shape	Output Shape	Configuration	Params
1	MS-Conv Block 1	Parallel Conv1D (3 branches) + CBAM	(B, 4, 1024)	(B, 64, 512)	K = (64, 32, 16), S = 2; CA ratio = 8	25,624
2	Max Pooling 1	MaxPool1D	(B, 64, 512)	(B, 64, 256)	K = 2, S = 2	0
3	MS-Conv Block 2	Parallel Conv1D (3 branches) + CBAM	(B, 64, 256)	(B, 128,128)	K = (64, 32, 16), S = 2; CA ratio = 8	283,928
4	Max Pooling 2	MaxPool1D	(B, 128, 128)	(B, 128, 64)	K = 2, S = 2	0
5	Conv Block 3 + CBAM	Conv1D + BatchNorm + CBAM	(B, 128, 64)	(B, 256, 32)	K = 8, S = 2, P = 3; CA ratio = 8, SA K = 7	295,168
6	Max Pooling 3	MaxPool1D	(B, 256, 32)	(B, 256, 16)	K = 2, S = 2	0
7	Global Avg Pooling	AdaptiveAvgPool1D	(B, 256, 16)	(B, 256, 1)	Output size = 1	0
8	Bottleneck Layer	FC + BN + Dropout (0.5)	(B, 256)	(B, 256)	Hidden dim = 256	66,304
9	Classifier	Fully Connected	(B, 256)	(B, 4)	Classes = 4	1028
10	Multi-Linear Mapping	Randomized Projection	$\left(B,\;256\right)⊗\left(B,\;4\right)$	(B, 1024)	*R_f_*: 256 × 1024, *R_g_*: 4 × 1024	0 *
11	Domain Discriminator	3-layer MLP	(B, 1024)	(B, 2)	Hidden = (1024, 1024, 2)	2,102,274

**Notes:** The MS-ACDAN with 4-channel input and sequence length of 1024; K, S, P, and CA denote kernel size, stride, padding, and channel attention ratio, respectively; B represents batch size; * Fixed random projection matrix (non-trainable); MS-Conv: Multi-Scale Convolution; CBAM: Convolutional Block Attention Module; BN: Batch Normalization; FC: Fully Connected; SA: Spatial Attention.

**Table 2:** Experimental conditions.

States	Working Conditions		
	Working Condition 1	Working Condition 2	Working Condition 3
Normal state	1 MPa	1.5 MPa	2 MPa
Fault State1 (Internal Leakage)	1 MPa	1.5 MPa	2 MPa
Fault State2 (Partial Blockage)	1 MPa	1.5 MPa	2 MPa
Fault State3 (Packing Leakage)	1 MPa	1.5 MPa	2 MPa

**Table 3:** Source-target domain configurations for the three transfer tasks.

Transfer Tasks	Source Domain	Target Domain	Characterization of Domain Shift
Task 1	A (1 MPa)	B (1.5 MPa)	Slight domain shift (small pressure difference)
Task 2	A (1 MPa)	C (2 MPa)	Severe domain shift (large pressure difference)
Task 3	B (1.5 MPa)	C (2 MPa)	Intermediate domain shift (medium pressure difference)

**Table 4:** The description of datasets.

Tasks	Working Conditions (MPa)	Fault	Label	Number of Samples
A	1	Normal	C1	249
		Internal Leakage	C2	249
		Partial Blockage	C3	249
		Packing Leakage	C4	249
B	1.5	Normal	C1	249
		Internal Leakage	C2	249
		Partial Blockage	C3	249
		Packing Leakage	C4	249
C	2	Normal	C1	249
		Internal Leakage	C2	249
		Partial Blockage	C3	249
		Packing Leakage	C4	249

**Table 5:** Ablation analysis of the MS-ACDAN framework.

Models	Tasks	Evaluation Metrics (%)			
		Accuracy	Precision	Recall	F_1_-Score
Raw-ACDAN	Task 1 (A → B)	84.25 ±1.86	81.14 ±2.03	83.54 ±1.92	82.81 ±2.01
	Task 2 (A → C)	68.31 ±3.15	68.50 ±2.78	69.74 ±2.63	69.12 ±2.71
	Task 3 (B → C)	81.70 ±2.52	81.25 ±2.16	82.04 ±2.00	81.63 ±1.98
MS-CDAN	Task 1 (A → B)	92.30 ±1.37	91.62 ±1.45	92.08 ±1.34	91.84 ±1.40
	Task 2 (A → C)	78.10 ±2.05	76.33 ±2.14	77.80 ±2.08	77.02 ±1.99
	Task 3 (B → C)	86.54 ±2.45	87.12 ±1.81	87.62 ±1.65	87.32 ±1.72
MS-ADANN	Task 1 (A → B)	91.50 ±1.42	90.74 ±1.50	91.25 ±1.48	90.90 ±1.40
	Task 2 (A → C)	76.82 ±2.26	75.19 ±2.38	76.44 ±2.23	75.74 ±2.30
	Task 3 (B → C)	86.54 ±1.79	85.84 ±1.87	86.24 ±1.74	86.05 ±1.81
Proposed	Task 1 (A → B)	95.84 ±0.90	95.21 ±1.04	95.60 ±0.98	95.42 ±1.02
	Task 2 (A → C)	82.50 ±1.68	80.94 ±1.80	82.10 ±1.74	81.53 ±1.68
	Task 3 (B → C)	91.39 ±1.25	90.80 ±1.35	91.02 ±1.28	90.92 ±1.23

**Notes:** Raw-ACDAN indicates the variant using raw vibration signals without CEEMDAN preprocessing; MS-CDAN indicates the variant removing channel attention; MS-ADANN indicates the variant replacing CDAN with vanilla DANN; Arrows denote migration tasks (e.g., A → B, A → C, B → C), representing the transfer from one operating condition to another.

## Data Availability

Data are available from the corresponding author upon reasonable request.
